# Deciphering motor dysfunction and microglial activation in mThy1-*α*-synuclein mice: a comprehensive study of behavioral, gene expression, and methylation changes

**DOI:** 10.3389/fnmol.2025.1544971

**Published:** 2025-02-13

**Authors:** Brett A. McGregor, Md. Obayed Raihan, Afrina Brishti, Junguk Hur, James E. Porter

**Affiliations:** ^1^Department of Biomedical Sciences, University of North Dakota School of Medicine and Health Sciences, Grand Forks, ND, United States; ^2^Department of Pharmaceutical Sciences, Chicago State University School of Pharmacy, Chicago, IL, United States

**Keywords:** microglia, synucleinopathy, epigenetics, DNA methylation, RNA-seq, alpha-synuclein, systems biology, transcriptome

## Abstract

**Introduction:**

Growing recognition of microglia’s role in neurodegenerative disorders has accentuated the need to characterize microglia profiles and their influence on pathogenesis. To understand changes observed in the microglial profile during the progression of synucleinopathies, microglial gene expression and DNA methylation were examined in the mThy1-*α*-synuclein mouse model.

**Methods:**

Disease progression was determined using behavioral tests evaluating locomotor deficits before DNA and RNA extraction at 7 and 10 months from isolated microglia for enzymatic methyl-sequencing and RNA-sequencing.

**Results:**

Pathway analysis of these changes at 7 months indicates a pro-inflammatory profile and changes in terms related to synaptic maintenance. Expression and methylation at both 7 and 10 months included terms regarding mitochondrial and metabolic stress. While behavior symptoms progressed at 10 months, we see many previously activated pathways being inhibited in microglia at a later stage, with only 8 of 53 shared pathways predicted to be directionally concordant. Despite the difference in pathway directionality, 21 of the 22 genes that were differentially expressed and annotated to differentially methylated regions at both 7 and 10 months had conserved directionality changes.

**Discussion:**

These results highlight a critical period in disease progression, during which the microglia respond to *α*-synuclein, suggesting a transition in the role of microglia from the early to late stages of the disease.

## Introduction

Synucleinopathy disorders refer to a group of complex neurodegenerative diseases, including Parkinson’s disease, Multiple System Atrophy, and Dementia with Lewy Bodies (Van der Perren et al., 2020). A small, soluble protein, *α*-synuclein, which normally functions in neurotransmitter release and synaptic vesicle trafficking at the neuronal synapse, instead misfolds and aggregates into insoluble clumps to form pathological structures known as Lewy bodies which have been linked with neurodegeneration (Gómez-Benito et al., 2020). In synucleinopathies, this degeneration is mainly located in the dopaminergic systems, resulting in a progressive loss of dopamine-producing neurons in areas such as the substantia nigra. This loss in the dopaminergic system leads to motor deficit symptoms such as bradykinesia, rigidity, and resting tremors, which are characteristic features of synucleinopathy disorders (Savica et al., 2019).

While pathological hallmarks primarily involve the loss of dopaminergic neurons, recent evidence suggests that microglia play a critical role in the pathogenesis of synucleinopathies (Li et al., 2023). Microglia are motile immune cells of the central nervous system (CNS) and play a crucial role in supporting neuronal function and protecting the CNS (Umpierre and Wu, 2021; Borst et al., 2021; Nebeling et al., 2023). The involvement of microglia in the aggregation, spreading, and clearance of misfolded proteins, including *α*-synuclein, highlights them as a key player in neurodegenerative diseases (Li et al., 2024; Cai et al., 2022; George et al., 2019; Scheiblich et al., 2021; Li et al., 2023).

Many symptoms of synucleinopathies, such as the propagation of α-synuclein, inclusion of the misfolded protein in microglia, inflammatory phenotypes, and significant motor function impairment, are also reported in mouse models of synucleinopathies (Tanriöver et al., 2020). These observations indicate the conserved role microglia likely play in neurodegeneration (McGregor et al., 2021; Bido et al., 2021a; Richter et al., 2023). A deeper exploration into the mechanisms regulating these systems in the presence of aberrant proteins may reveal novel therapeutic targets for a spectrum of neuroinflammatory and degenerative conditions. Experiments conducted using the mThy1-*α*-synuclein model of synucleinopathy in this study suggest α-synuclein inclusion in microglia and neurons as well as an increased proportion of activated microglial over time when compared to littermate wild-type controls (Watson et al., 2012; Tanriöver et al., 2020).

Microglia phenotypes can be differentiated utilizing gene expression patterns, and our previous study reported significantly different gene expression and methylation patterns in microglia from synucleinopathy mice at both an early and advanced stage of pathology (McGregor et al., 2021). Changes to the cellular transcriptome and methylome play a significant role in regulating cellular function and are vital in understanding cellular contributions to disease pathogenesis (Hadad et al., 2019; Zhao et al., 2022). Alterations in the microglial transcriptome and methylome have previously been investigated to understand their contribution to the cause of various neurodegenerative diseases (Prater et al., 2023; Hunter et al., 2021). However, the influence of *α*-synuclein on gene expression and regulation has not been specifically examined to understand the role of microglia cells in the progression of synucleinopathies.

Our prior work examined the early (3-month) and late (13-month) stages of pathology, and the current study bridges these extremes. We hypothesize that profiling microglia during the mid-stages of the disease will reveal transcriptome and methylome changes relating to the developing disease phenotype at 7 and 10 months. The selection of 7- and 10-month time points was guided by previous studies demonstrating that motor dysfunction and neuroinflammatory changes in synucleinopathy models tend to occur during mid-disease stages. In particular, studies using mThy1-*α*-synuclein Line 61 mice have reported early motor impairments starting around 5 months and progressing through 9 months, making 7- and 10-month time points ideal for capturing key phenotypic transitions (Rabl et al., 2017; Kim et al., 2015; Fleming et al., 2004; Valera et al., 2015; Fleming et al., 2008). These time points are also expected to capture dynamic changes in microglial activation and proliferation, which have been shown to fluctuate as *α*-synuclein pathology evolves. Therefore, 7 and 10 months were chosen to provide insights into the progression of synucleinopathy-related changes at a critical stage between early and late disease. Shifts in the cellular transcriptome and methylome during pathological progression hold significant importance in disease research and guide the development of targeted therapeutics (Hunter et al., 2021). Identifying key regulatory mechanisms will provide critical insights into the disease process and drive future mechanistic and preclinical-translational research.

To test this hypothesis, we employed our previously characterized mThy1-*α*-synuclein mice to distinguish distinct transcriptome and methylome changes and to identify candidate genetic and epigenetic factors regulating the behavior of microglia in synucleinopathies, which could pave the way to targeted therapeutics. With our modified microglia purification protocol (Raihan et al., 2021), we generated *α*-synuclein-responsive microglial transcriptomic and DNA methylation profiles from 7- and 10-month cohorts with or without synucleinopathy-related motor impairment. The data collected were also used to identify dysregulated pathways when compared between cohorts. In addition to pathways related to inflammation and mitochondrial dysfunction, described previously in the early and advanced stages of our model, we uncovered microglial-associated pathways that may give additional insight into synucleinopathy pathogenesis. The present study provides insight into synucleinopathy-related changes in microglia gene expression and methylation profiles and describes pathway-level changes associated with pathological progression.

## Materials and methods

### Mice/animals use

Male wildtype and mThy1-*α*-synuclein mice were procured from the Chesselet laboratory at the University of California, Los Angeles. The corresponding background of mThy1-α-synuclein mice is the B6D2F1/J mice (stock# 100006) from The Jackson Laboratory (Bar Harbor, ME). In all performed studies, 7- and 10-month-old male mice were used to avoid any adverse effects of the females’ ability to inactivate X chromosome expression, which contains the inserted human α-syn gene (Fleming et al., 2004). The genotype of all mice was verified with PCR analysis of tail snip DNA via general end-point PCR (McGregor et al., 2021). Animals were housed under standard laboratory conditions on a 12-h light/dark cycle with free access to food and water. Mice were euthanized in a CO2 chamber (small mouse cage) with a flow rate of 3–5 liter(s) per minute for 2–5 min. The chamber was cleaned between uses and euthanasia was confirmed via paw pinch. All experiments were carried out according to the National Institute of Health Guide for the Care and Use of Laboratory Animals (N.I.H. Publications No. 80-23, revised 1978). The Institutional Animal Care and Use Committee (IACUC) approved the study protocol (IACUC Protocol #: 2004-7).

### Behavioral assessment

#### Behavior tests

All motor symptom assessments were performed in the University of North Dakota Behavioral Research Core Facility (BRCF). We performed open field, pole test, foot printing for stride length, hind-limb grasping, inverted grid test, and body weight measurement. Body weight was measured before the start of behavioral testing at the early phase of the light cycle. Animals underwent training for the pole test on two consecutive days, with the final test performed on day 3 alongside all other tests. On test day, the open field test was performed before allowing animals to rest for around 1 h and then testing on the pole test. The following day, hind-limb grasping and inverted grid tests were performed after foot printing.

#### Pole test

The pole test was used to assess motor coordination and balance in the mice (Giraldo et al., 2018). Mice were placed head up near the top of a 1 cm diameter and 50 cm long pole, which was positioned vertically on a soft platform. Mice were allowed to descend to the base freely while the time to descend the entire length of the pole was recorded. A maximum cutoff time of 60 s was provided to complete the tasks. The longest time for each individual mouse over three consecutive test trials was recorded.

#### Inverted wire/wire hang test/inverted grid test

The inverted grid test, also known as the mesh grip test, assesses grip strength and balance (Giraldo et al., 2018). Mice were placed in the center of the wire mesh (30 cm by 30 cm screen with 1 cm wide mesh) and held horizontally. The screen was then inverted head-over-tail and placed 40 cm above an open cage with soft padding. The time until the mouse fell off the screen or remained within the cutoff time of 60 s was recorded.

#### Open field test

An open-field test was used to quantify general locomotion (Uddin et al., 2021). Mice were placed in an open enclosure (40.6 cm × 40.6 cm × 38.1 cm, San Diego Instruments, San Diego, CA, USA) and allowed to explore freely over 10 min after 30 s of acclimation. The free movements of the individual mouse were recorded with a digital video camera placed directly on top from the middle of the open enclosure (C525 HD webcam, Logitech International, Newark, CA, USA). The behavioral parameters such as total distance traveled, velocity, and time spent in the 20 cm × 20 cm center zone were automatically analyzed using the ANY-maze software (Stoelting Co., Wood Dale, IL, USA).

#### Body weight

The mice were transferred to the behavioral research core facility 3–4 weeks prior to behavioral testing at 7- & 10- months of age. Before starting the behavioral testing, body weight was recorded in grams.

#### Footprinting/stride length

The stride length test was used to measure walking ability, muscular strength, and balance. Stride length is the distance between successive points of initial contact of the same foot with shorter stride lengths characteristic of a Parkinsonian gait (Wan et al., 2021). The fore- and hind paws of each mouse were painted with nontoxic red and black ink, respectively. Mice were placed on a narrow path covered with white paper and allowed to walk freely from one end to the other. At least three distances between two footprints on each side of the white paper with color footprints were measured using a ruler for stride length calculation. For each measurement, the first and last footsteps were excluded. The trial was repeated if the mouse stopped in the middle of the tunnel.

#### Hind-limbs clasping score

The hind-limb clasping test is used to measure the severity of motor dysfunction (Sampson et al., 2020). Mice were gently lifted upward by their tail for 30 s and the clasping score was assigned based on the following criteria: 0- was assigned to mice where both hind-limbs extended outwards for the majority of the observation time, 1- was assigned to mice where one hind-limb was retracted for at least half of the observation time, 2- was given to mice where both hind-limbs were partially retracted for more than half of the observation time, and 3- was assigned to mice where both hind-limbs were retracted completely for more than half of the observation time.

#### Statistical analysis

Behavioral data are represented as mean ± S.E.M. and were analyzed by one-way ANOVA and the Holm-Sidak *post hoc* test using GraphPad Prism v10.3.1 (GraphPad Software, Inc., USA).

#### Purification of microglia and extraction of DNA–RNA

Following our earlier published protocol, microglia were isolated from a single mouse whole brain for dual DNA and RNA extraction (Raihan et al., 2021). Initially, six animals in each group were assessed for behavioral changes associated with disease progression; however, four samples (two 7-month controls, one 10-month control, and one 10-month Thy1-*α*-synuclein mouse) did not yield sufficient RNA for follow-up sequencing. In brief, dissected brain tissue was dissociated using the Neural Tissue Dissociation Kit (Miltenyi Biotech). Following myelin debris and Red Blood Cell removal using myelin debris and RBC removal solutions (Miltenyi Biotech), microglia cells were labeled using MACS CD11b magnetic (Microglia) micro-beads (Miltenyi Biotech). CD11b magnetic-labeled and activated microglia cells were then sorted using double miniMACS MS filter column held inside the miniMACS magnet attached to the MACS separator. Due to the limited RNA/DNA yield and the risk of compromising sample viability, microglial cell counts were not recorded during this study. However, based on previous experience with this methodology, the typical yield of microglial cells per sample in this model were 3.0–5.0×10^5^ CD11b positive cells (Raihan et al., 2021). Microglial purity was estimated to be 90–95% based on the protocol used. The proportion of microglia relative to other brain cells was not measured. Based on previous studies, microglia are estimated to represent approximately 2.1% of total brain cells (Miller et al., 2017). DNA and RNA from isolated microglia were extracted from 7- and 10-month-old mThy1-*α*-synuclein mice and wild-type littermate controls using the Qiagen Dual RNA/DNA miniprep kit (Raihan et al., 2021). Both wildtype and mThy1-α-synuclein mice at each timepoint were processed together from euthanasia to nucleic acid isolation.

### RNA-sequencing and bioinformatic processing

Isolated RNAs (minimum RIN value ≥8.0) were submitted to the Yale Genomics Core for sequencing. All samples were sequenced in a single run with approximately 60 million 150 bp paired-end read pairs obtained for each sample using Illumina NovaSeq 6,000. The resulting reads were subject to quality assessment of RNA-Seq FASTQ files using FastQC version 0.11.9 (Wingett and Andrews, 2018). Raw reads were trimmed using Trim Galore version 0.6.7 to remove adapters, low-quality reads, and reads less than 40 bp in length (Felix Krueger et al., 2023). Cleaned reads were mapped to the mm10 mouse reference genome using HISAT2 version 2.2.1 (Kim et al., 2019). The featureCounts function from Rsubread version 2.18.0 assigned mapped read to unique genomic features (Liao et al., 2019), allowing for the identification of differentially expressed genes (DEGs) using DESeq2 version 1.44.0 with a significance cutoff of <0.05 Benjamini-Hochberg adjusted *p*-value (Love et al., 2014).

### Enzymatic methyl sequencing and bioinformatic processing

Isolated DNAs were submitted to the Yale Genomics Core for Enzymatic methyl-seq (EM-seq). Nucleotides were prepared using the NEBNext enzymatic methyl-seq kit for 350 million 150 bp paired-end read pairs per sample to be sequenced on an Illumina NovaSeq 6,000. Samples were split into two batches for sequencing, but no batch effect was detected. EM-seq reads were first assessed for quality using FastQC version 0.11.9 (Wingett and Andrews, 2018). Raw reads were trimmed using Trim Galore version 0.6.7 to remove adapters, low-quality reads, and reads below 40 bp in length (Felix Krueger et al., 2023). Cleaned reads were mapped to the mm10 mouse reference genome using Bismark version 0.23.1 and bowtie2 version 2.2.5 (Langmead and Salzberg, 2012). Uniquely mapped reads were assessed for changes in methylation between wild-type and mThy1-*α*-synuclein mice using methylKit version 1.22.0 (Akalin et al., 2012). Differentially methylated regions (DMRs) were determined using a 1,000 bp sliding window with a 25% minimum difference in cytosine methylation levels between groups with q-value <0.01. The q-value, in this context, is an adjusted *p*-value calculated using the sliding linear model (SLIM) in methylKit. The Genomation R package was used to annotate DMRs, based on genes and CpG island features 2, 5, and 10 kb regions upstream from transcription start sites (Akalin et al., 2015). CpG island and other regional annotations were obtained from the University of California Santa Cruz (UCSC) Genome Browser (Nassar et al., 2023).

#### Functional enrichment analysis

Both DEGs and gene-associated DMRs were assessed for functional enrichment analysis using our in-house R package richR^1^ and the Gene Ontology biological processes (GO-BP) annotation (Thomas et al., 2022). Enriched biological functions and pathways within our gene lists were determined based on a cutoff of Benjamini-Hochberg adjusted p-value <0.05. These results were visualized using richR as a dot plot where dot size indicates the number of genes in our dataset annotated to those functions and dot color represents the significance based on a −log10(adj. *p*-value) scale.

Identified DEGs were also assessed using QIAGEN Ingenuity Pathway Analysis (IPA) for canonical pathway enrichment (Kramer et al., 2014). DEGs were subject to core analysis within IPA, while using their biological filters for nervous system tissue and mouse species. Pathways with a −log(*p*-value) > 1.3 were considered significantly enriched. Z-scores, based on observed gene expression changes, were calculated within IPA to provide directionality context to pathway changes in terms of activation or inhibition. Pathway figures were generated through the use of QIAGEN IPA (QIAGEN Inc., https://digitalinsights.qiagen.com/IPA). The distribution of DEGs involved in enriched IPA pathways was visualized using the EnhancedVolcano R package^2^ (Blighe and Lewis, 2018). While the complete DEG list is available in the scatter plot, the pathway network may not incorporate all DEGs either to optimize visualization or because their role may not be known.

## Results

### 7- and 10-month-old, mThy1-*α*-synuclein mice gain less body weight with increasing age and display motor impairments

**Figure 1:**
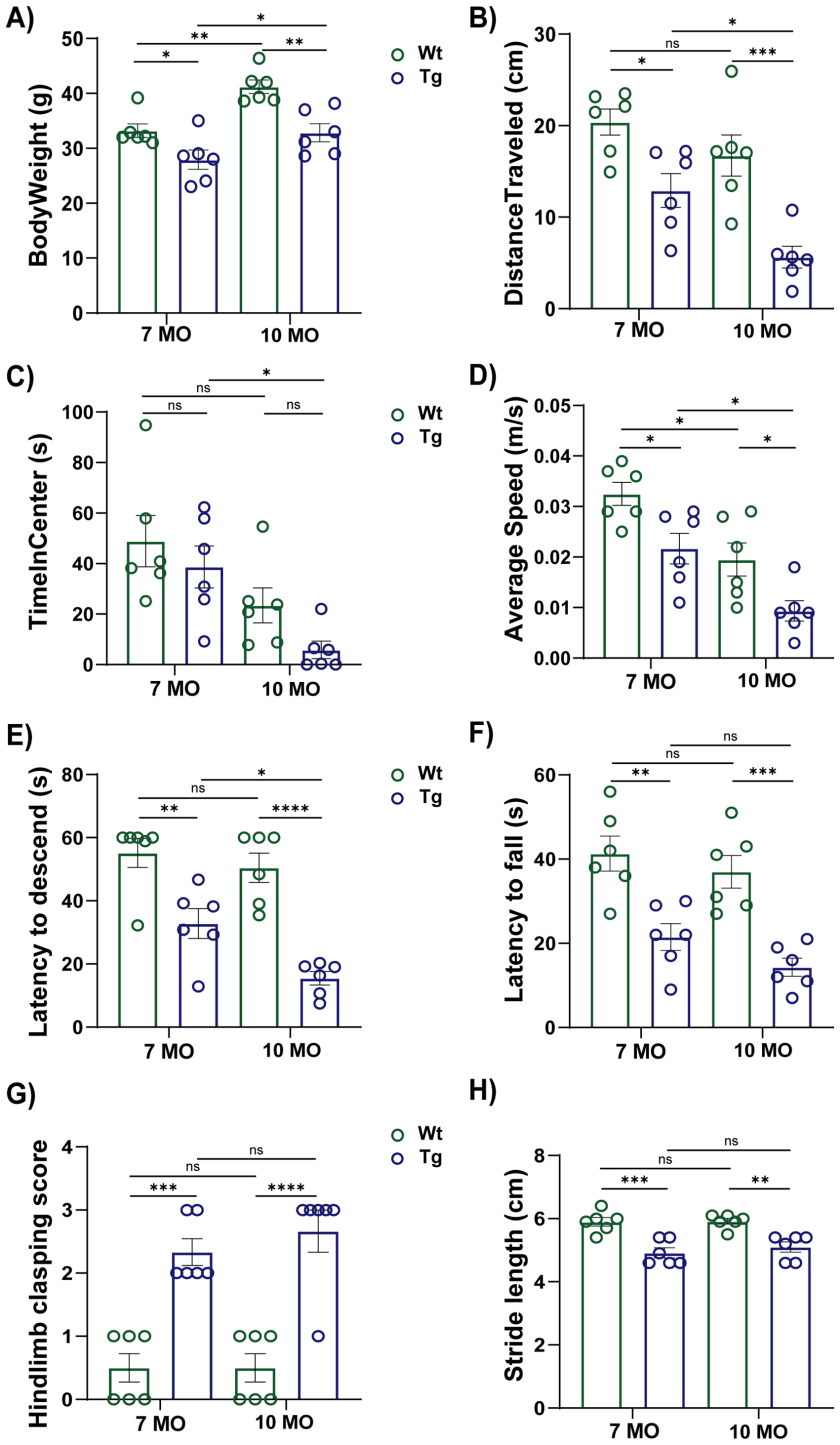
7- and 10-month-old male, Thy1-α-synuclein mice have increased weight loss and deficits in motor symptoms. In body weight measurement **(A)**, body weight (g). In the open field test: total distance traveled (cm) **(B)**, time spent in center (s) **(C)**, and average movement velocity (cm/s) **(D)**. In the pole descend test **(E)**, latency to descend the entire length of the vertical pole (s), with Tg mice exhibiting a significant difference in latency to fall compared to control mice. In the inverted grid test **(F)**, latency to fall off the screen (s). In the hind-limb clasping test **(G)**, hind-limb clasping severity scores. In the footprint test **(H)**, stride length (cm). Data are presented as mean ± S.E.M. (*n* = 6 mice/group). *p < 0.05, **p < 0.01, ***p < 0.001.

In motor performance and body weight assessment, both 7- and 10-month-old *α*-synuclein mice showed significant changes. As shown in Figure 1A, 7-month-old mThy1-α-synuclein mice displayed a lower body weight than non-transgenic wild-type control mice, and the difference became more significant at 10 months. In the open field test, *α*-synuclein mice at both time points traveled a shorter total distance (Figure 1B), however, time spent in the center (Figure 1C) was not significantly different compared to age-matched littermate wild-type control. While α-synuclein mice spent less time in center compared to their wild-type littermates, this difference was not statistically significant which could be attributed to the use of the standard open field test or to the possibility that age-specific brain regions affected by synucleinopathy pathology may not directly translate to anxiety. The movement velocity of the *α*-synuclein mice at both time points was also lower than observed in control mice (Figure 1D). The track plots from the open field test also demonstrate different movement patterns between α-synuclein and control mice. In the pole test (Figure 1E), α-synuclein mice at both time points showed shorter latencies to descend from the vertical pole than the littermate control mice. As shown in Figure 1F, α-synuclein mice at both time points took less time to fall off the inverted wire screen than littermate controls. Wild-type control mice at both time points displayed a normal extension reflex. However, they showed a significantly lower hind-limb clasping severity score (Figure 1G) when compared with α-synuclein mice. Gait abnormalities were tested by painting the paws of each mouse, and α-synuclein mice at both time points had a shorter forelimb stride length with higher variation (Figure 1H) compared to littermate wild-type control mice. These behavioral data are in agreement with earlier data from mouse models of α-synucleinopathies (Giraldo et al., 2018; Rabl et al., 2017; Chesselet et al., 2012). Significant motor deficits in five distinct behavioral tests confirm symptoms of synucleinopathy in our subject animals. The observed phenotype facilitates our investigation into the neuroinflammatory status of these animals, specifically their microglia profiles, and how these may be associated with disease progression.

### Gene expression and methylation changes associated with mThy1-α-synuclein overexpression at 7 months

**Figure 2:**
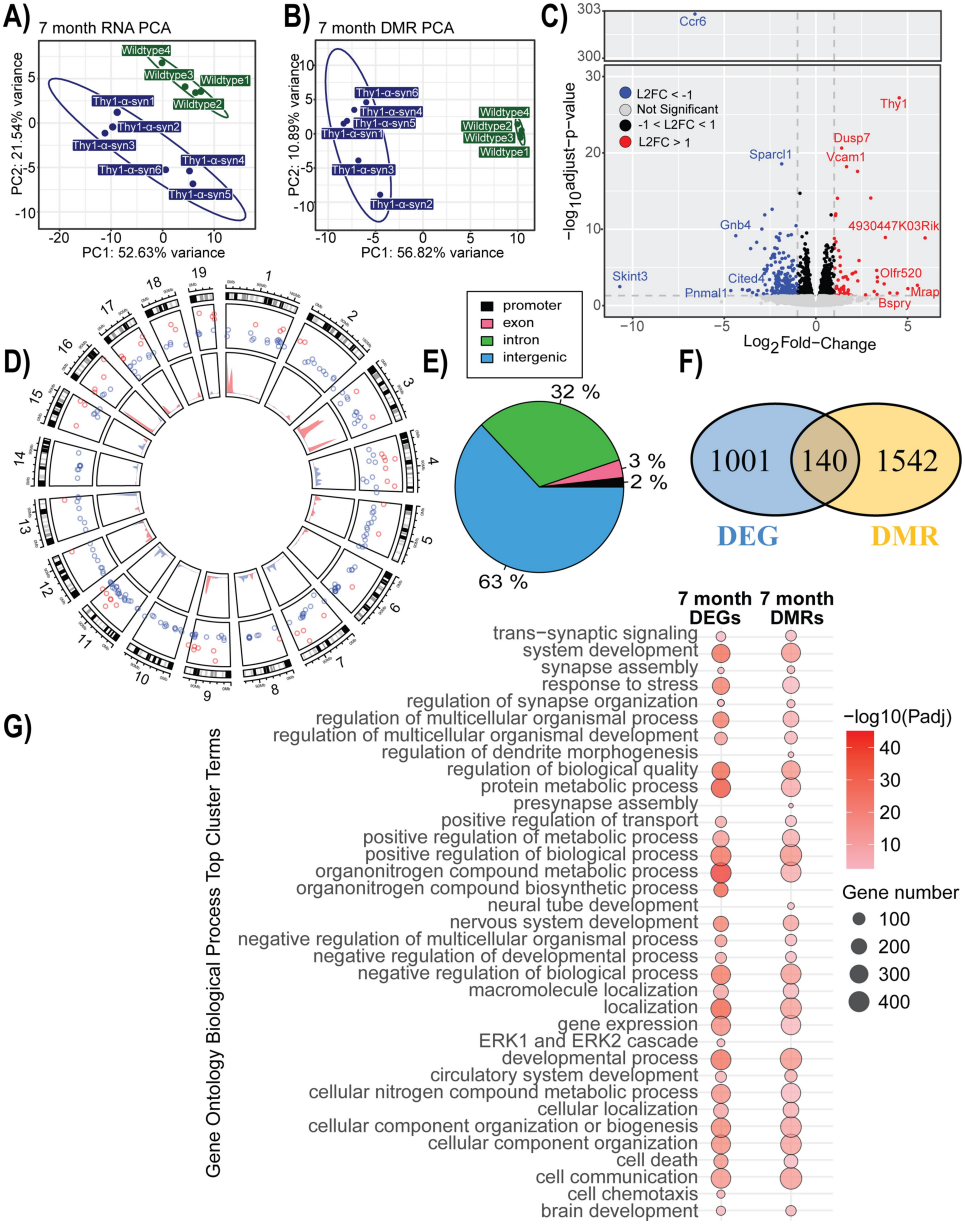
Gene expression and methylation changes associated with mThy1-α-synuclein overexpression at 7 months. **(A,B)** Principal component analysis (PCA) of gene expression and methylation profiles. **(C)** Volcano plot with differentially expressed genes (DEGs) highlighted in blue (down-regulated) and red (up-regulated). Genes with the most significant changes are labeled. **(D)** Circle plot showing the distribution of significant differentially methylated regions (DMRs) on the inner track, along with DEGs in the outer track. Colors show the direction of change, with blue representing a negative change and red showing an increase when compared to wild-type controls. **(E)** Genomic location distribution of DMRs. **(F)** A Venn diagram showing the overlap between DMRs and DEGs. **(G)** A dot plot illustrating the top 20 significantly enriched Gene Ontology (GO) biological processes terms enriched within our DEGs and DMRs. The dot color represents enrichment significance, while the dot size indicates the number of associated genes.

Following the behavior phenotyping of our animals, the brain was extracted, and microglia were isolated for RNA-sequencing and enzymatic DNA methylation sequencing to assess gene expression and regulation changes occurring in this critical cell population during disease progression. Principal component analysis (PCA) showed a distinct grouping of our samples based on their genotype in terms of both RNA expression and patterns of DNA methylation (Figures 2A,B). Mice at 7 months of age had 1,141 DEGs when comparing transgenic samples to control, with 52% of the genes being down-regulated (Figure 2C; Supplementary Table S1). There were 6,712 identified DMRs with the largest changes observed being increases in methylation on chromosomes 1, 3, 9, and 16 and decreased methylation on chromosomes 4, 7, 8, 11, and 13 (Figure 2D; Supplementary Table S2). The majority of significant DMRs were annotated to intergenic and intronic regions (Figure 2E). DMRs, annotated to genes based on the UCSC genome browser annotation, were associated with 140 DEGs. Shared genes, as well as complete DEG and DMRs, were separately assessed for functional enrichment (Figure 2F; Supplementary Tables S3, S4). The common DEG and DMR genes were enriched in GO terms related to homeostasis, metabolism, and regulation. Many top cluster terms were enriched in both the transcriptome and methylome in the 7-month-old mice. The unique terms identified based on the DEG list were related to signaling and immune recruitment, along with terms related to chemotaxis and ERK1/2 cascades. Terms only enriched from the DMR data were related to development, morphogenesis, and synapse-related pathways (Figure 2G; Supplementary Tables S5, S6).

**Figure 3:**
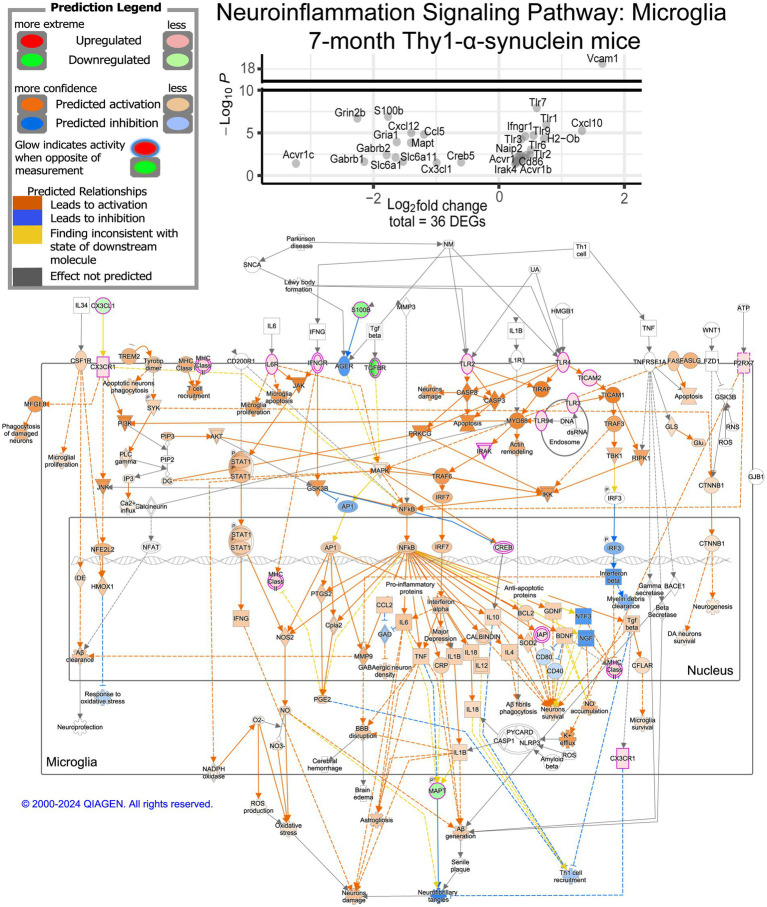
Neuroinflammation signaling pathway activity and member DEGs from mThy1-α-synuclein overexpression at 7 months. Identified DEGs at 7 months were submitted to Qiagen’s Ingenuity Pathway Analysis (IPA) for core analysis and enrichment of canonical pathways. DEGs from our data involved in determining the enrichment of this pathway are demonstrated in the dot plot with significance levels based on a −log10(adjusted *p*-value), and the x-axis represents the observed log2 fold change. Measured DEGs within the pathway are outlined in purple and colored either red for up-regulated or green for down-regulated. IPA leverages observed changes to calculate a Z-score to predict activation (orange) or inhibition (blue) of multiple elements of the pathway. Yellow interactions represent inconsistent downstream measurements compared to the calculated prediction while any gray interactions could not be predicted.

We further explored the pathways represented within our DEGs using IPA, which predicts directionality using a Z-score based on expression values. While GO annotation provides a broad view of our overall results, IPA’s predicted activity offers deeper insight into the observed changes. Overall, 141 pathways were significantly enriched in IPA’s annotation (Supplementary Table S7). Among the significantly enriched pathways, neuroinflammation signaling stood out, encompassing several pathways of interest within the data (Figure 3). Notably, seven of the thirty-six DEGs leading to this pathway’s enrichment are TLR genes, which have been linked to *α*-synuclein-induced inflammation (Fellner et al., 2013). The majority of the neuroinflammation signaling pathway is predicted to be activated at 7 months, including reactive oxygen species (ROS) production and phagocytosis, while the major inflammatory pathway predicted is NF-ĸB signaling (Supplementary Figure S1). Toll-like-receptor signaling was also represented in the data, indicating a possible IL-1-induced apoptosis signal (Supplementary Figure S2). Pyroptosis signaling was also enriched, possibly indicating high stress levels in the microglia at 7 months (Supplementary Figure S3). Another related pathway of interest is the Parkinson’s signaling pathway (Supplementary Figure S4). Within this pathway, the microglia inflammatory pathways are activated, and it further indicates mitochondria dysfunction likely associated with α-synuclein.

### Gene expression and methylation changes associated with mThy1-α-synuclein overexpression at 10 months

**Figure 4:**
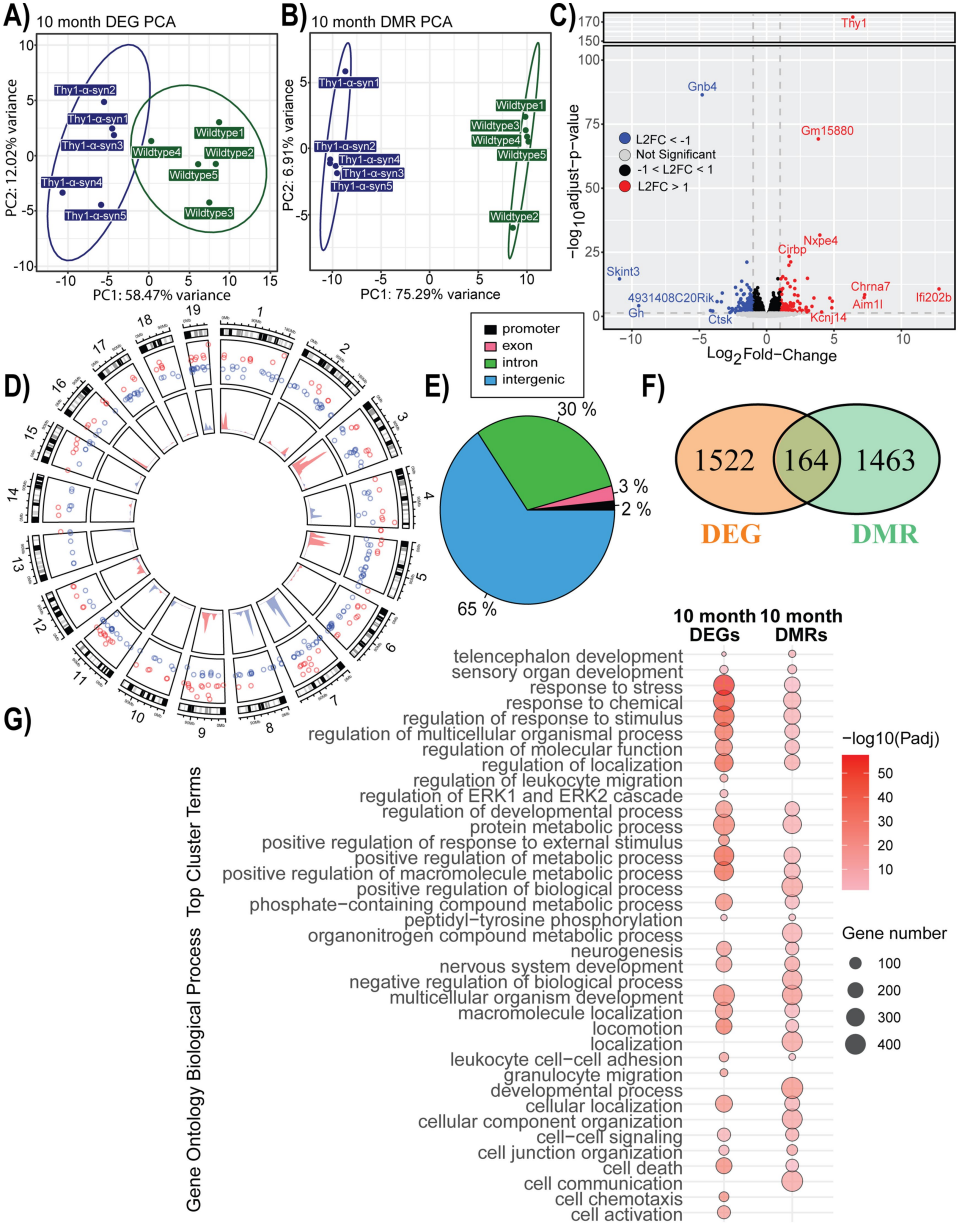
Gene expression and methylation changes associated with mThy1-α-synuclein overexpression at 10 months. **(A,B)** Principal component analysis (PCA) of gene expression and methylation profiles. **(C)** Volcano plot with differentially expressed genes (DEGs) highlighted in blue (down-regulated) and red (up-regulated). Genes with the most significant changes are labeled. **(D)** Circle plot showing the distribution of significant differentially methylated regions (DMRs) on the inner track, along with DEGs in the outer track. Colors show the direction of change, with blue representing a negative change and red showing an increase compared to wild-type controls. **(E)** Genomic location distribution of DMRs. **(F)** A Venn diagram showing the overlap between DMRs and DEGs. **(G)** A dot plot illustrating the top 20 significantly enriched Gene Ontology (GO) biological processes terms enriched within our DEGs and DMRs. The dot color represents enrichment significance, while the dot size indicates the number of associated genes.

Mice were similarly phenotyped and analyzed for DEGs and DMRs between transgenic and wild-type groups at 10 months of age. PCA plots show that samples were grouped based on their genotype. However, the DMR data separated more clearly than DEGs, which were more similar between groups (Figures 4A,B). We identified 1,686 DEGs between α-synuclein and control mice, with 52% being down-regulated (Figure 4C; Supplementary Table S8). Between α-synuclein and control mice, 8,531 DMRs were identified, and similarly to methylation changes at 7 months, we observed increased methylation on chromosomes 1, 3, 9, and 16 along with decreases in methylation levels on chromosomes 4, 7, 8, 11, and 13 (Figure 4D; Supplementary Table S9). Additional changes that were more evident at 10 months included increased methylation on chromosomes 5 and 12 as well as decreased methylation on chromosome 19. The identified DMRs are primarily associated with intergenic (65%) and intronic (30%) regions (Figure 4E). Following DMR annotation to genes, we found 164 genes, which were both differentially expressed and associated with DMRs within our data (Figure 4F; Supplementary Table S10). Both complete lists of DEGs and DMRs, as well as the shared genes, were individually assessed for functional enrichment (Figure 4G; Supplementary Tables S11–S13). Functions uniquely associated with identified DEGs were often related to immune recruitment. Terms enriched in DMRs were mainly related to development, process regulation, and cell communication. Common genes differentially methylated and expressed were enriched in GO terms related to stress response and cellular organization. The majority of identified cluster terms overlap between transcriptome and methylome, although there are more data type unique terms at 10 months than identified at 7 months.

**Figure 5:**
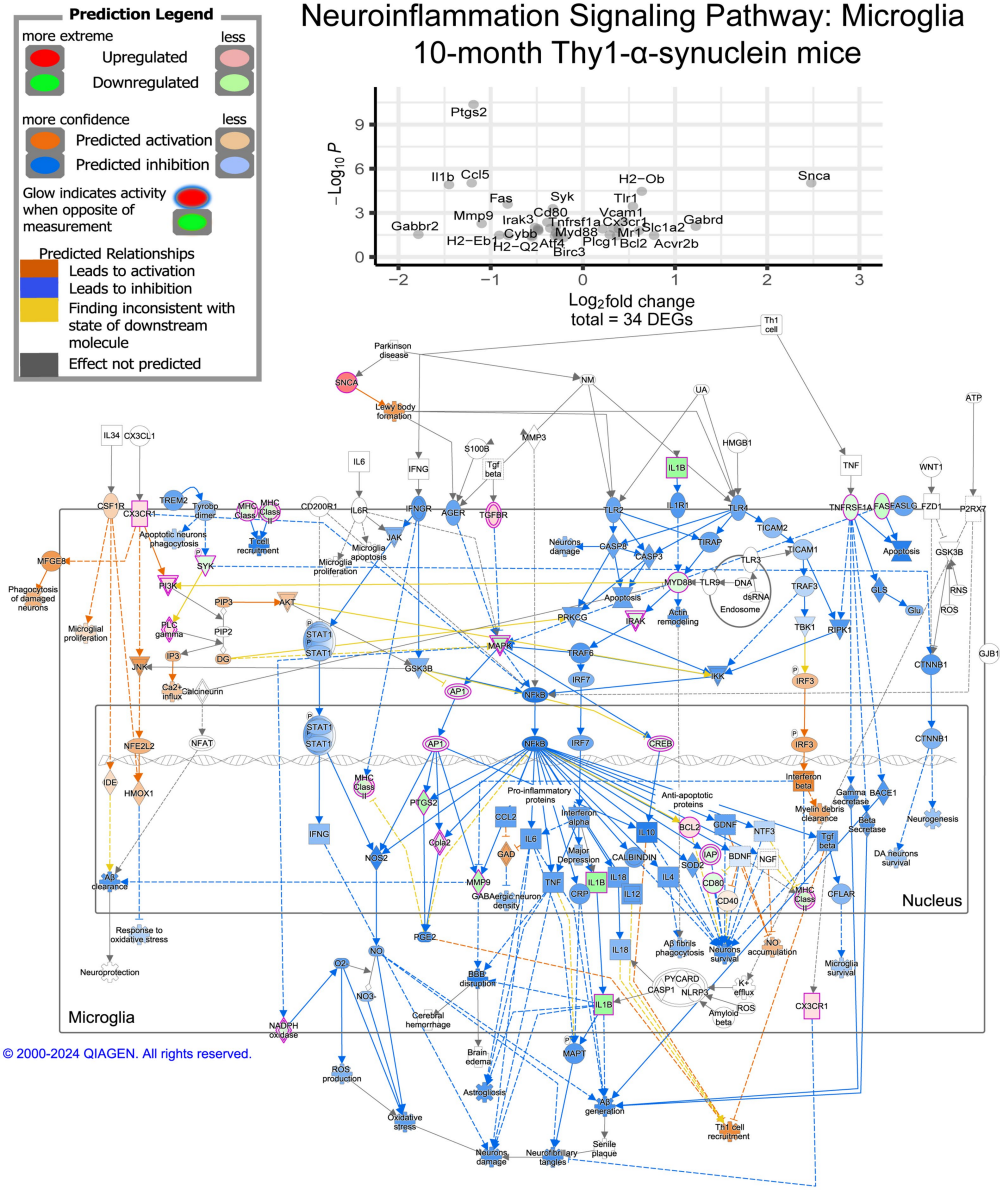
Neuroinflammation signaling pathway activity and member DEGs from mThy1-α-synuclein overexpression at 10 months. Observed DEGs at 10 months were submitted to Qiagen’s Ingenuity Pathway Analysis (IPA) for core analysis and enrichment of canonical pathways. Identified DEGs determining enrichment of this pathway are plotted on the dot plot with significance levels based on a −log10(adjusted p-value) while the x-axis represents the observed log2 fold change. Measured DEGs within the pathway are outlined in purple and colored either red for up-regulated or green for down-regulated. IPA leverages observed changes to calculate a Z-score useful for predicting activation (orange) or inhibition (blue) within the pathway. Yellow interactions represent inconsistent downstream measurements when considering the calculated prediction while any gray interactions could not be predicted.

The observed gene expression changes were also examined using IPA to determine additional details, such as the directionality of changes, and to facilitate a deeper understanding of the transition between 7 and 10 months associated with the progression of the behavioral phenotype. Overall, 187 canonical pathways within IPA were above our −log10(*p*-value) threshold for significance (Supplementary Table S14). When compared to our IPA results at 7 months, 53 pathways are enriched in both time points, although only 8 of these pathways are concordant between timepoints according to Z-score (Supplementary Figure S5; Supplementary Table S15). The neuroinflammation signaling pathway is one shared pathway between timepoints that exhibits dramatic changes in directionality from 7 to 10 months in these animals (Figure 5). While phagocytic activity is still predicted to be activated, the majority of the signaling pathways related to proinflammation appear to be inhibited based on our transcription data. The Parkinson’s signaling pathway was also significantly enriched at 10 months and demonstrated a likely down-regulation of the pro-inflammatory pathways within microglia (Supplementary Figure S6). Additional elements of the pathway related to oxidative stress and mitochondria dysfunction are predicted to be activated at this later time point, which may indicate accumulating stress and damage as the model phenotype advances. We see similar changes from activation to inhibition in the pyroptosis signaling pathway, such as inhibition of the pro-inflammatory response and inflammasome formation (Supplementary Figure S7).

### Shared gene expression and methylation changes at 7 and 10 months

**Figure 6:**
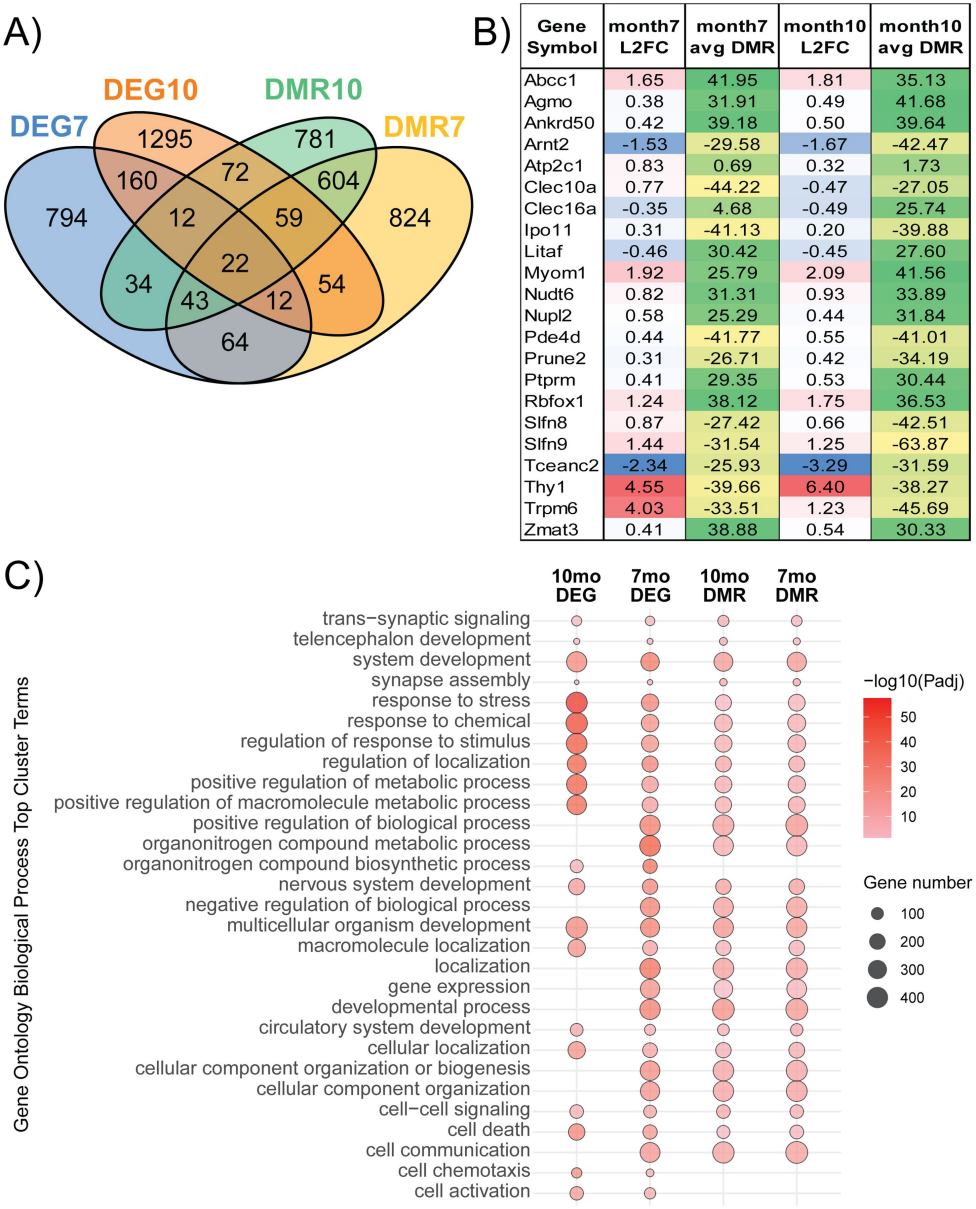
Shared gene expression and methylation changes at 7 and 10 months. **(A)** Genomic changes in gene expression or DNA methylation were examined for overlap across modalities and the study timepoints. **(B)** Out of the total genes changed across our four datasets, 22 genes were significant in all four. The log2 fold-change (L2FC) and the difference in methylation levels (%) between mThy1-α-synuclein over-expressers and wild-type control mice of these genes are available in the table. **(C)** Enrichment analysis of each dataset using Gene Ontology (GO) annotation was performed to identify biological processes (BP) overrepresented within our gene list. Significantly enriched processes were clustered to reduce redundancy, and the top 10 cluster terms for each dataset are included in the dot plot. The dot color represents enrichment significance, while the dot size provides information on the number of genes identified within our data, which are annotated to each term.

Collected data were also examined for shared and distinct DEGs and DMR-associated genes at 7 and 10 months (Figure 6A). There were 22 genes shared across all four datasets collected, which have 95% concordance in both directions of gene expression and methylation changes to associated DMRs (Figure 6B). Furthermore, the amount of change between groups is often increased from 7 to 10 months. *Clec10a* was the only shared gene, which changed directionality between 7 and 10 months, going from up-regulated to down-regulated. *Trpm6*, unlike other shared genes, had a noticeably smaller log2 fold-change at 10 months than at 7, although it was up-regulated at both timepoints. Changes in methylation for shared genes followed a similar pattern of concordance. These data were evaluated for shared and distinct functional enrichment using GO-BP (Figure 6C). Many of the terms relating to metabolic regulation, development, and response to stress/stimulus were enriched at both time points and included in both DEG and DMR lists. Other terms were uniquely enriched by data modality rather than timepoint, such as chemotaxis and cell activation, which were enriched in the transcriptome data but were not included in the methylation data at either 7 or 10 months. Interestingly, no terms were unique to either timepoint, but the DMR datasets for both timepoints were similarly enriched across these top cluster terms. The transcriptome-related terms that were no longer significant at 10 months, as they were at 7, are related to terms such as localization, developmental process, and cellular component organization.

## Discussion

Our current study aims to further investigate *α*-synuclein reactive microglia in the mThy1-α-synuclein model by capturing additional timepoints to shed light on the transition state between the early and late stages previously examined (McGregor et al., 2021). Transgenic mice demonstrated a pathological phenotype related to motor impairment compared to littermate wild-type controls. Gene expression and DNA methylation changes in microglia occurring during disease progression provide unique insight into their dynamic role in balancing defense and collateral damage.

The 7- and 10-month timepoints were selected based on observed synucleinopathy-associated motor deficient phenotypes. There is a general consensus that the alteration of *α*-synuclein biology leads to motor dysfunction and body weight reduction (Giraldo et al., 2018; Rabl et al., 2017; Chesselet et al., 2012). The behavior tests employed here are associated with motor dysfunction and disease progression in several models of neurodegeneration (Roshanbin et al., 2021; Zhu et al., 2016). More specifically, the hind-limb clasping reflex is a measure of striatal dysfunction (Zhang et al., 2014), and increased hind-limb clasping severity has been reported to be associated with severe inflammation and axon injury (Cahill et al., 2019). Transgenic mice at both time points showed overt changes in body weight, grip strength, hind-limb clasping, motor activity, and gait abnormalities. Our current behavioral data further validates earlier findings about motor impairments in synucleinopathies and supports the possible correlation between abnormal α-synuclein accumulation, microglial activation, and motor deficit phenotypes (Stoyka et al., 2020). Comparison of transgenic mice with littermate wild-type controls at both 7- and 10-month time points revealed significant body weight loss and movement deficits in the transgenic mice, with these phenotypes worsening markedly at 10-months. However, other behaviors, such as latency to descend and time spent in the center of the open field, remained relatively stable between these time points, suggesting that certain motor deficits may plateau as the disease progresses.

Gene expression and methylation changes from the microglia within these animals provide unique insight into their cellular efforts during disease progression. Examining identified DEGs using GO enrichment analysis revealed that the transcriptome and methylome changes observed in 7-month-old animals represented a variety of pathways mainly involved in metabolism and development. While changes in mitochondria and, consequently metabolism have been linked to *α*-synuclein aggregation, the development pathways may be related to microglia’s role in synaptic maintenance or reflect morphological changes (Lurette et al., 2023; Doorn et al., 2014; Austin et al., 2011). Many of the significantly enriched immune and inflammatory terms cluster within the response to stress term (Supplementary Table S5). When considering the immune response terms in context with chemotaxis and recruitment functions there is a clear inflammatory response at 7 months in the microglia from transgenic animals.

Exploring these pathways using IPA suggests a TLR-mediated response considering eight different TLRs are differentially expressed at 7 months (Supplementary Table S1). TLRs have been linked by multiple studies to the *α*-synuclein induced immune response (Venezia et al., 2017; Shao et al., 2019; Choi et al., 2020; Kim et al., 2013; Kim et al., 2016; Fellner et al., 2013). There are multiple ways for TLR signaling to induce inflammation, which were significantly enriched in our data, such as NF-ĸB, Nod1/2, and Trem1 (Supplementary Figures S1, S8, S9). These pathways were all predicted by IPA to activate immune response, suggesting many microglia are in a pro-inflammatory state at 7 months. Another important point to consider is that the top three most significant IPA pathways at 7 months include initiation, elongation, and termination of eukaryotic translation, which all have a negative z-score, suggesting that protein synthesis from messenger RNA is inhibited.

Further analysis of key pathways involved in microglial activation and proliferation, such as NF-κB signaling, Toll-like receptor signaling, and pyroptosis, demonstrates the evolving role of microglia during disease progression. At 7 months, pro-inflammatory pathways are highly active, contributing to the neuroinflammatory environment observed in synucleinopathy. By 10 months, these same pathways are inhibited, suggesting a shift in microglial behavior from a pro-inflammatory to a more regulated or protective state. This change underscores the dynamic balance between inflammation and neuroprotection as microglia respond to *α*-synuclein overexpression.

We expected a positive relationship between pro-inflammatory genes and symptom progression; instead, the DEG changes at 10 months often reflect inhibition of these same pathways. Both Nod1/2 and Trem1 signaling, while both significantly enriched in our 10-month data, are predicted to be inhibited, and NF-ĸB was not significant (Supplementary Figures S10, S11). One possible method of down-regulating the immune response could be the significant activation of LXR/RXR and PPAR*α*/RXRα, which are either down-regulated or not enriched at the 7-month timepoint (Supplementary Figure S12–S14). Although the inflammatory status at 10 months appears to be down-regulated, IPA predicted that phagocytosis elements of these neuroinflammatory pathways remain active. This could be expected since the transgenic animals continue to produce human α-synuclein. The high degree of concordance within the shared genes taken in context with the IPA pathway results suggests that multiple elements of the pathways are changing to influence directionality rather than a few centrally critical genes. This can also be observed when considering the low level of DEG overlap between similarly enriched IPA pathways at both timepoints. It will be important for future studies to maintain biological context to determine if external or internal signaling is responsible for these changes to the microglia’s inflammatory profile.

There is evidence that the microglia are overly stressed with cell death-related pathways such as pyroptosis and immunogenic cell death signaling being enriched and predicted to be activated at 7 months and inhibited at 10 months (Supplementary Figures S2, S7, S15). A recent study reported accelerated motor coordination impairments and loss of motor neurons in the substantia nigra associated with the depletion of microglia (Pereira et al., 2023). Another possible source of stress in the microglia could be related to metabolizing extracellular dopamine, which generates additional oxidative stress and possible mitochondrial damage. α-Synuclein has previously been shown to interact with tyrosine hydroxylase (TH) to regulate the production/metabolism of dopamine, and our data indicate differential expression of dopamine metabolism genes in 7/10-month “Line 61” mice (Venda et al., 2010). Dopamine receptors are also expressed in microglia, and a recent study in human patients demonstrated that a tonic level of dopamine improves movement by providing an implicit “motor motivational” signal (Gepshtein et al., 2014).

While the 10-month timepoint reveals a shift in microglia behavior, the prolonged pro-inflammatory state observed at earlier stages may have lasting consequences on disease pathology or even contribute to disease initiation. It’s likely initially beneficial for microglia to phagocytose *α*-synuclein aggregates but subsequent activation leads to an inflammatory cascade, secreting inflammatory cytokines such as TNF-α, IL-1*β*, IL-6, IL-1α, and reactive oxygen species which are known to have neurotoxic effects (Tewari et al., 2021; Hughes et al., 2019; Kim et al., 2013). Microglia driven inflammation is potentially a contributing cause of the neurodegeneration observed in synucleinopathies as reactive microgliosis in the substantia nigra has been observed months prior to the loss of dopaminergic neurons (Duffy et al., 2018). Microglia specific over expression of α-synuclein has also been shown *in vivo* to induce significant loss of dopaminergic neurons (Bido et al., 2021b). Activated microglia have been shown to weaken the blood brain barrier allowing peripheral immune cells to invade the CNS and further amplify neuroinflammation (Haruwaka et al., 2019). Treatment interventions targeting inflammatory pathways such as minocycline, glucocorticoids, and non-steroidal anti-inflammatory drugs (NSAIDs) have shown protective properties for dopaminergic neurons in certain models of neurodegenerative conditions such as Parkinson’s Disease. Minocycline has been shown to effectively protect dopaminergic neurons and decrease glia activation in lipopolysaccharide or 6-hydroxydopamine challenged mice but can exacerbate the loss of dopaminergic neurons in MPTP induced models (He et al., 2001; Tomas-Camardiel et al., 2004; Noble et al., 2009). Glucocorticoids such as dexamethasone and naloxone have also been shown to slow neuronal loss while decreasing glia activation and proinflammatory cytokine release (Castano et al., 2002; Kurkowska-Jastrzebska et al., 2004; Liu et al., 2000). Long-term NSAID use, specifically ibuprofen, also has been suggested as a therapeutic option by potentially reducing Parkinson’s disease risk by 21% although there have been contradicting findings from population and meta-analysis studies on this topic (Gao et al., 2011; Manthripragada et al., 2011; Gagne and Power, 2010). By reducing the loss of dopaminergic neurons many of these studies have also observed improved phenotypic behavior in animal models, suggesting both a critical role of microglia in disease onset and progression as well as candidates for therapeutic intervention. As we discuss the implications of our data in the context of published knowledge, it is important to recognize the limitations of the data generated by this study. Microglia were isolated from the whole brain and represented a diverse microglia population across all brain regions. While this likely means our data are from a diluted cell population rather than the specifically disease-associated microglia, it also implies that many disease-related changes may be more extreme than the values observed. Recent studies in Alzheimer’s disease have focused on microglia associated with amyloid-β plaques, and a similar technique may yield more specific information about synucleinopathies and *α*-synuclein aggregates (Wood et al., 2022; Walker et al., 2022; Chen et al., 2020). Another limitation is that our data are limited to microglia, while the brain and its immune response involve multiple cell types playing various roles. It would be necessary for future studies to capture all cell types within the context of their environment to discern more information about the critical players and the overall impact of inflammation on disease progression. Finally, our data are limited to measuring transcription-level changes based on RNA counts and assessing transcriptional regulation through DNA methylation. While protein-level data are more translatable, RNA-level studies often provide multiple clues and have the advantage of accurate and affordable high-throughput methods (Edfors et al., 2016).

Our findings indicate that the loss of microglia function, possibly through changes in gene expression, likely contributes to observed motor function deficits in synucleinopathy. In our model of synucleinopathy, in microglia, we observe regulatory changes indicating a decreased maintenance role and expression changes for pro-inflammatory signals when exposed to aberrant *α*-synuclein. The inflammatory environment likely increases the oxidative stress on an already vulnerable population of dopaminergic neurons. Collectively, our current work details the genetic effect of α-synuclein on the microglial transcriptome and provides a comprehensive resource for decoding microglial involvement in synucleinopathies, shedding light on how microglial function influences disease pathology and symptoms.

## Data Availability

The raw and processed DNA methylation (GSE275511) and RNA sequencing (GSE275510) are available through the Gene Expression Omnibus (GEO) database.

## Footnotes

^1^ github.com/guokai8/richR

^2^ github.com/kevinblighe/EnhancedVolcano
