# Supplementary material for: Deciphering motor dysfunction and microglial activation in mThy1-α-synuclein mice: a comprehensive study of behavioral, gene expression, and methylation changes
Source: Front Mol Neurosci. 2025 Feb 13;18:1544971. doi: 10.3389/fnmol.2025.1544971 (PMC11865073; doi:10.3389/fnmol.2025.1544971)
Supplement: Supplementary file 2 [file Data_Sheet_1.pdf]

A PDF file containing the full resolution images is available at
https://figshare.com/s/074233fe6debb7951c43

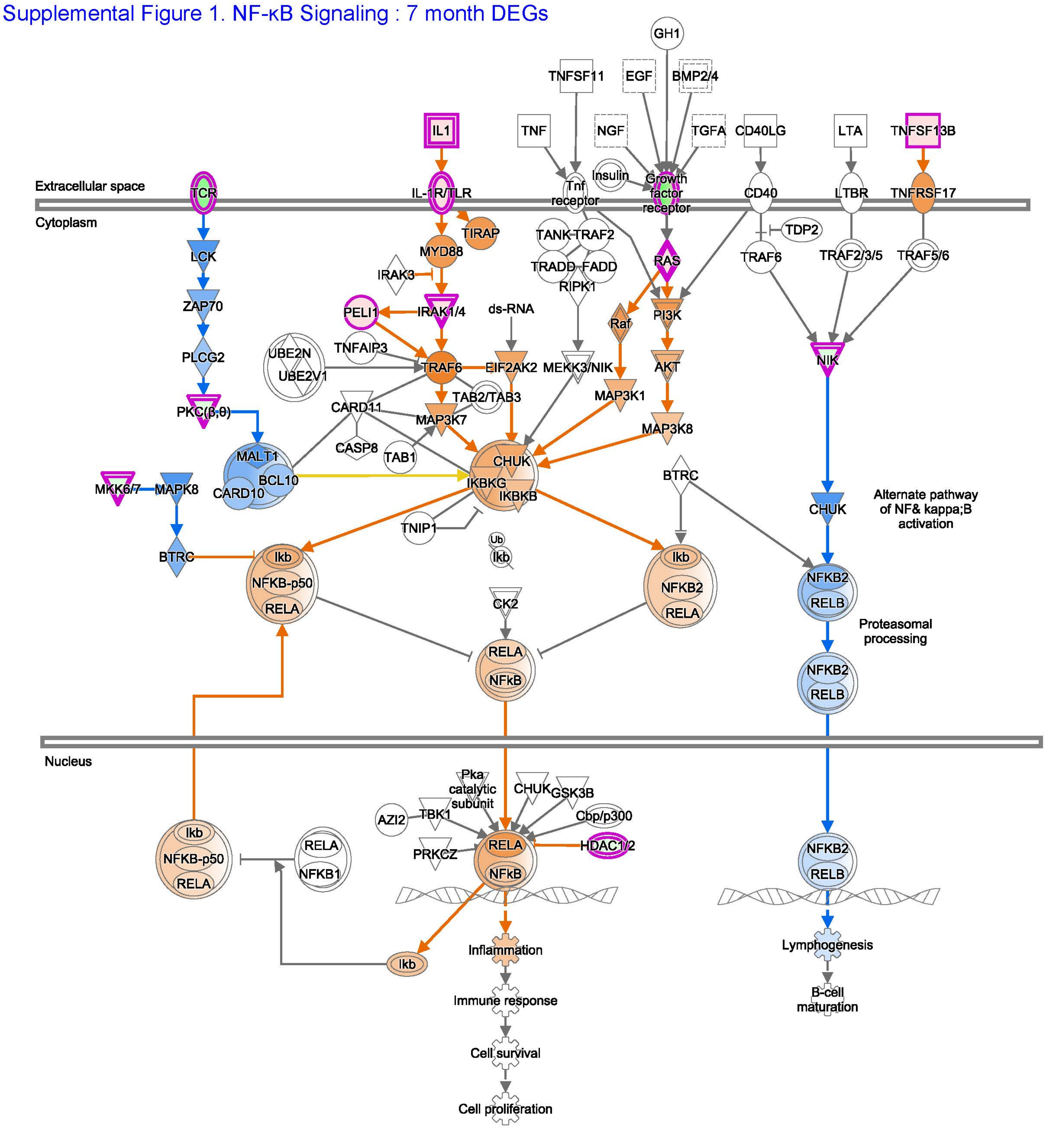

© 2000-2024 QIAGEN. All rights reserved.

Supplemental Figure 1. Ingenuity Pathway Analysis of 7-month Gene Expression: Enrichment of NF-κB Signaling. Significant differentially expressed genes (DEGs) at 7 months were submitted to Qiagen's Ingenuity Pathway Analysis (IPA) for core analysis and enrichment canonical pathways. NF-κB Signaling represents a key regulatory pathway for innate and acquired immunity and response to stress. Identified DEGs involved in the pathway are outlined in purple and colored red if they are upregulated or green for down regulated genes. IPA also calculates a Z score for the pathway and individual interactions based on observed expression values to predict activation (orange) or inhibition (blue). Gray interactions could not be predicted, and yellow interactions may be inconsistent between predicted and observed values. The data at 7 months predicts the NF-κB signaling pathway to be activated based on gene expression levels of toll-like receptor and downstream kinases.

Supplemental Figure 2. Toll-like Receptor Signaling : 7 month DEGs

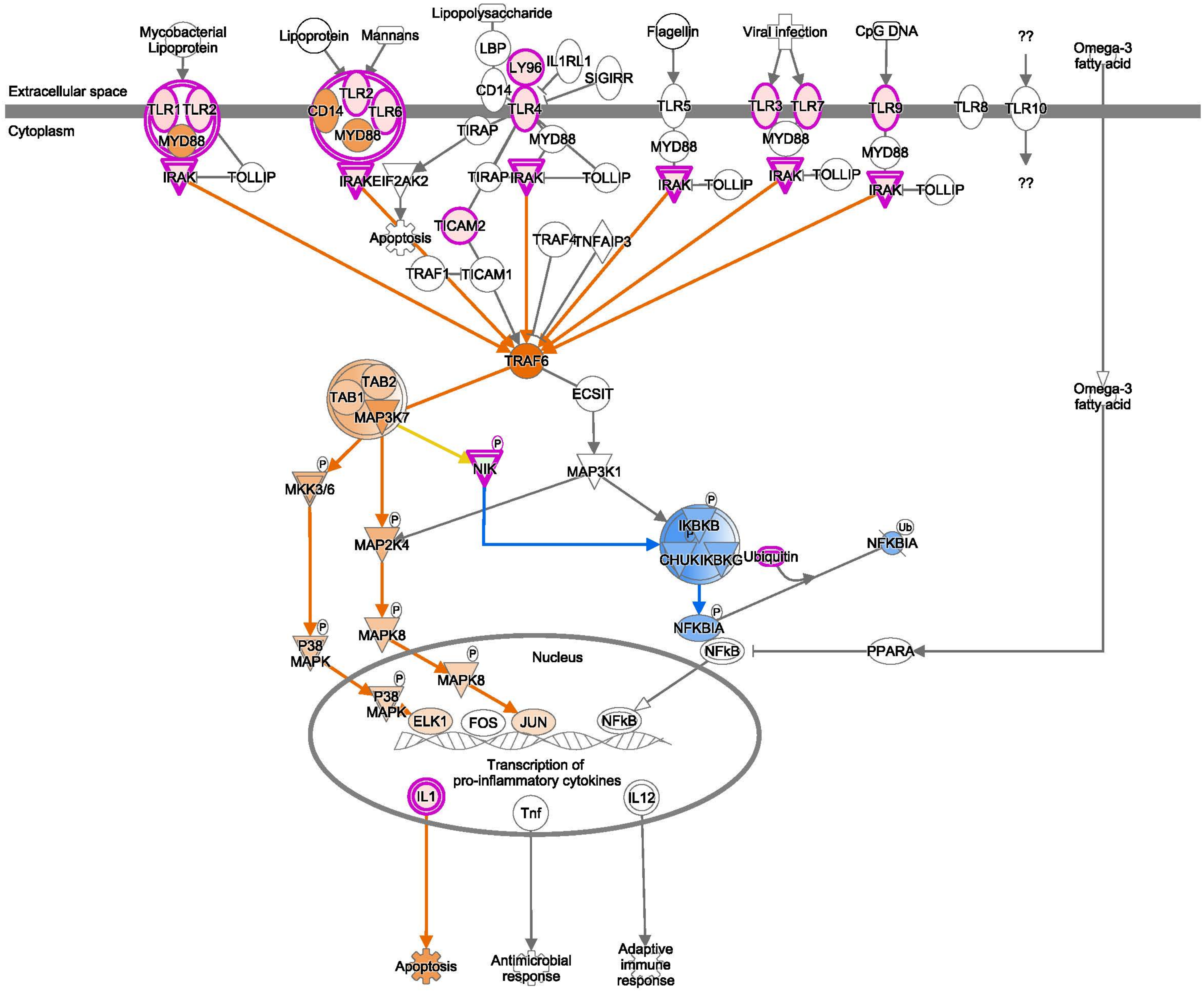

© 2000-2024 QIAGEN. All rights reserved.

Supplemental Figure 2. Ingenuity Pathway Analysis of 7-month Gene Expression: Enrichment of Toll-like Receptor Signaling. Significant differentially expressed genes (DEGs) at 7 months were submitted to Qiagen's Ingenuity Pathway Analysis (IPA) for core analysis and enrichment canonical pathways. Toll-like receptors (TLRs) are pathogen-associated pattern recognition receptors often responsible for activating the immune response by identifying structurally conserved elements of microbes. Identified DEGs involved in the pathway are outlined in purple and colored red if they are upregulated or green for down regulated genes. IPA also calculates a Z score for the pathway and individual interactions based on observed expression values to predict activation (orange) or inhibition (blue). Gray interactions were not able to be predicted and yellow interactions may be inconsistent between predicted and observed values. IPA likely predicts activation at the pathway level based on increased expression of multiple TLRs and downstream elements involving IL-1.

Supplemental Figure 3. Pyroptosis Signaling Pathway : 7 month DEGs

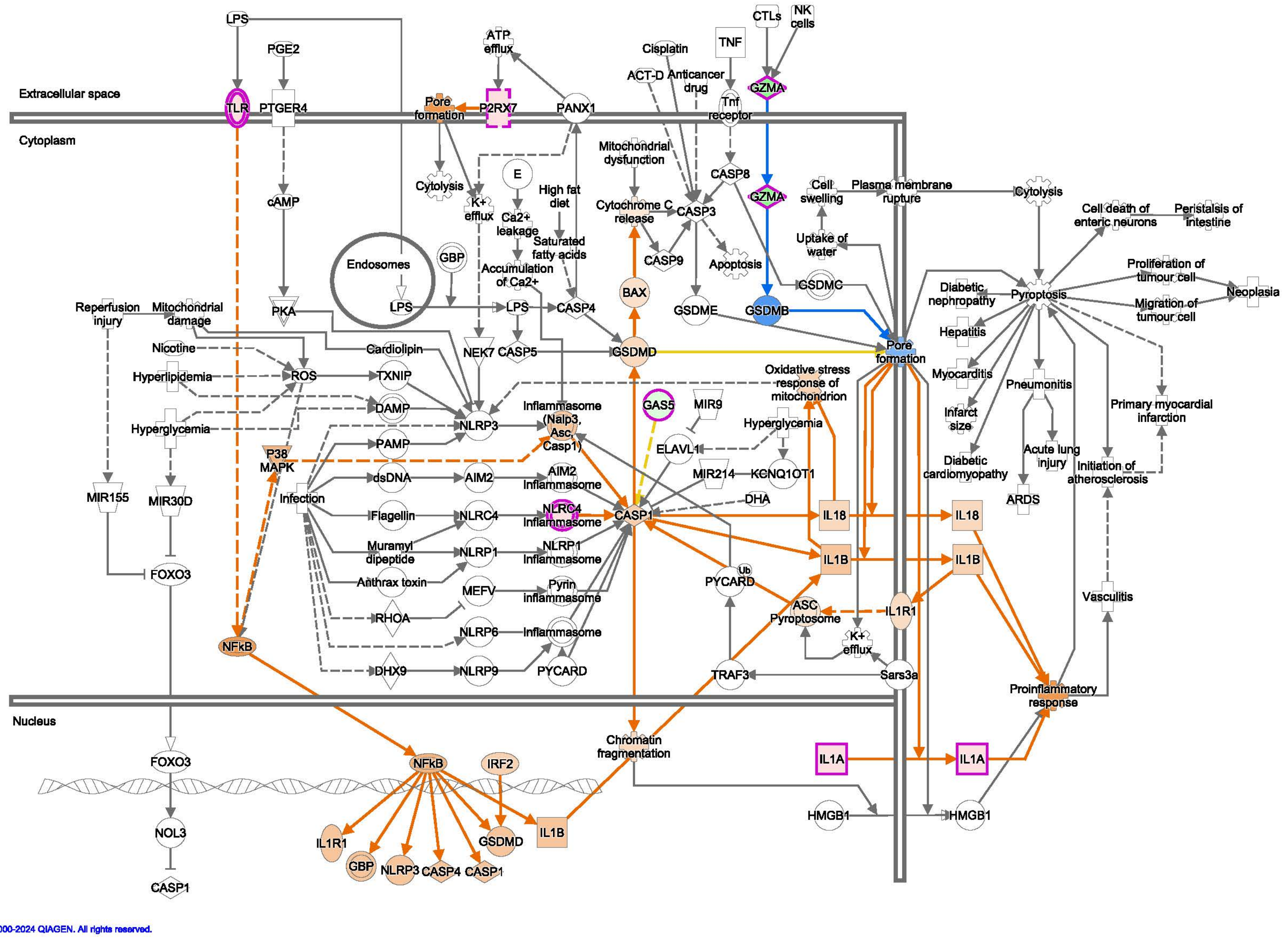

© 2000-2024 QIAGEN. All rights reserved.

Supplemental Figure 3. Ingenuity Pathway Analysis of 7-month Gene Expression: Enrichment of Pyroptosis Signaling Pathway. Significant differentially expressed genes (DEGs) at 7 months were submitted to Qiagen's Ingenuity Pathway Analysis (IPA) for core analysis and enrichment canonical pathways. Identified DEGs involved in the pathway are outlined in purple and colored red if they are upregulated or green for down regulated genes. IPA also calculates a Z score for the pathway and individual interactions based on observed expression values to predict activation (orange) or inhibition (blue). Gray interactions were not able to be predicted and yellow interactions may be inconsistent between predicted and observed values. IPA likely predicts activation at the pathway level based on increased expression of NLRC4 inflammasome, TLRs, and IL1A.

Supplemental Figure 4. Parkinson's Signaling Pathway : 7 month DEGs

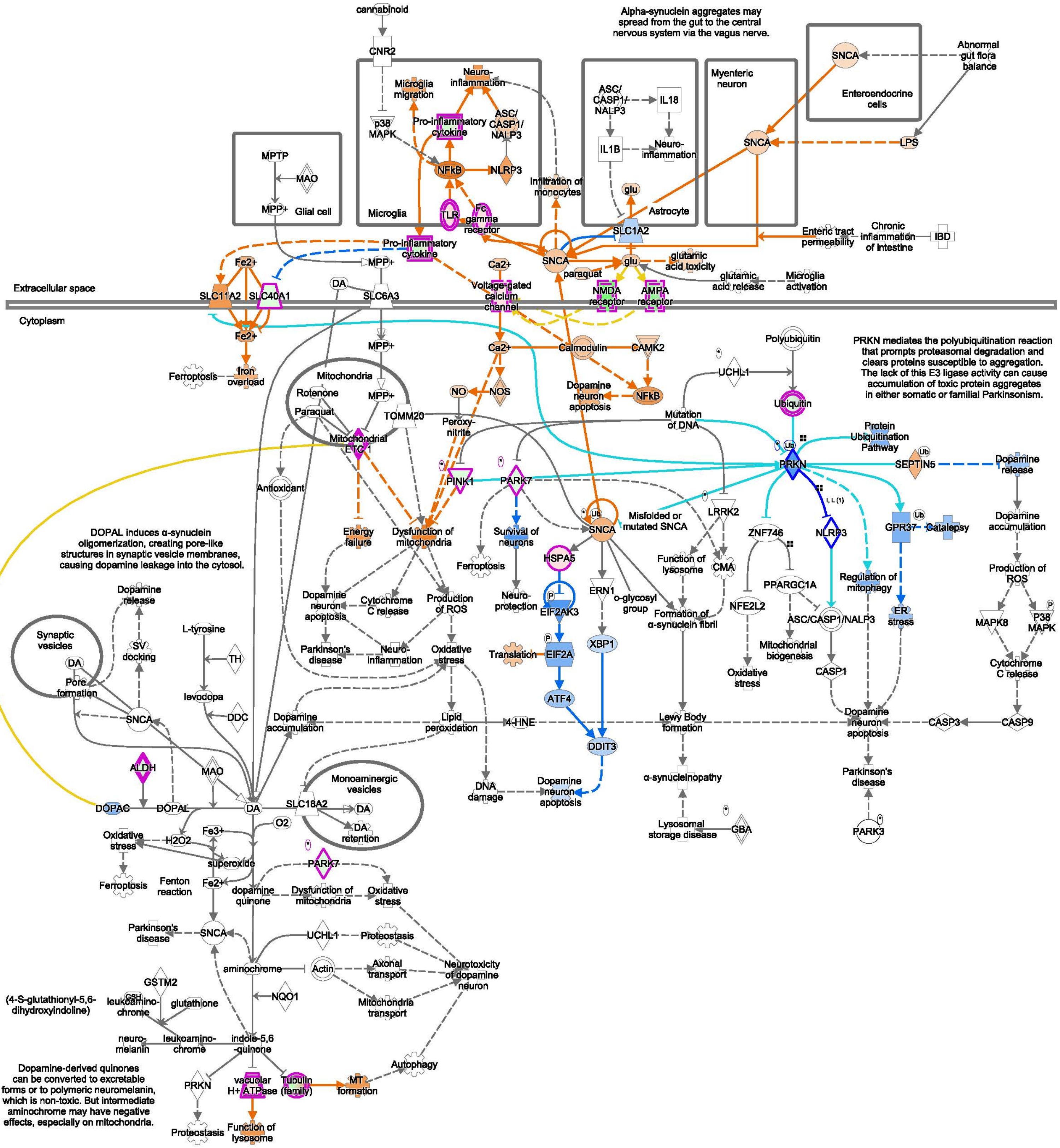

© 2000-2024 QIAGEN. All rights reserved.

Supplemental Figure 4. Ingenuity Pathway Analysis of 7-month Gene Expression: Enrichment of Parkinson's Signaling Pathway. Significant differentially expressed genes (DEGs) at 7 months were submitted to Qiagen's Ingenuity Pathway Analysis (IPA) for core analysis and enrichment canonical pathways. Identified DEGs involved in the pathway are outlined in purple and colored red if they are upregulated or green for down regulated genes. IPA also calculates a Z score for the pathway and individual interactions based on observed expression values to predict activation (orange) or inhibition (blue). Gray interactions were not able to be predicted and yellow interactions may be inconsistent between predicted and observed values. IPA likely predicts activation at the pathway level based on changes to Parkinson's associated genes such as PINK1 and PARK7 along with high level changes such as mitochondria disruption or increased inflammatory activity.

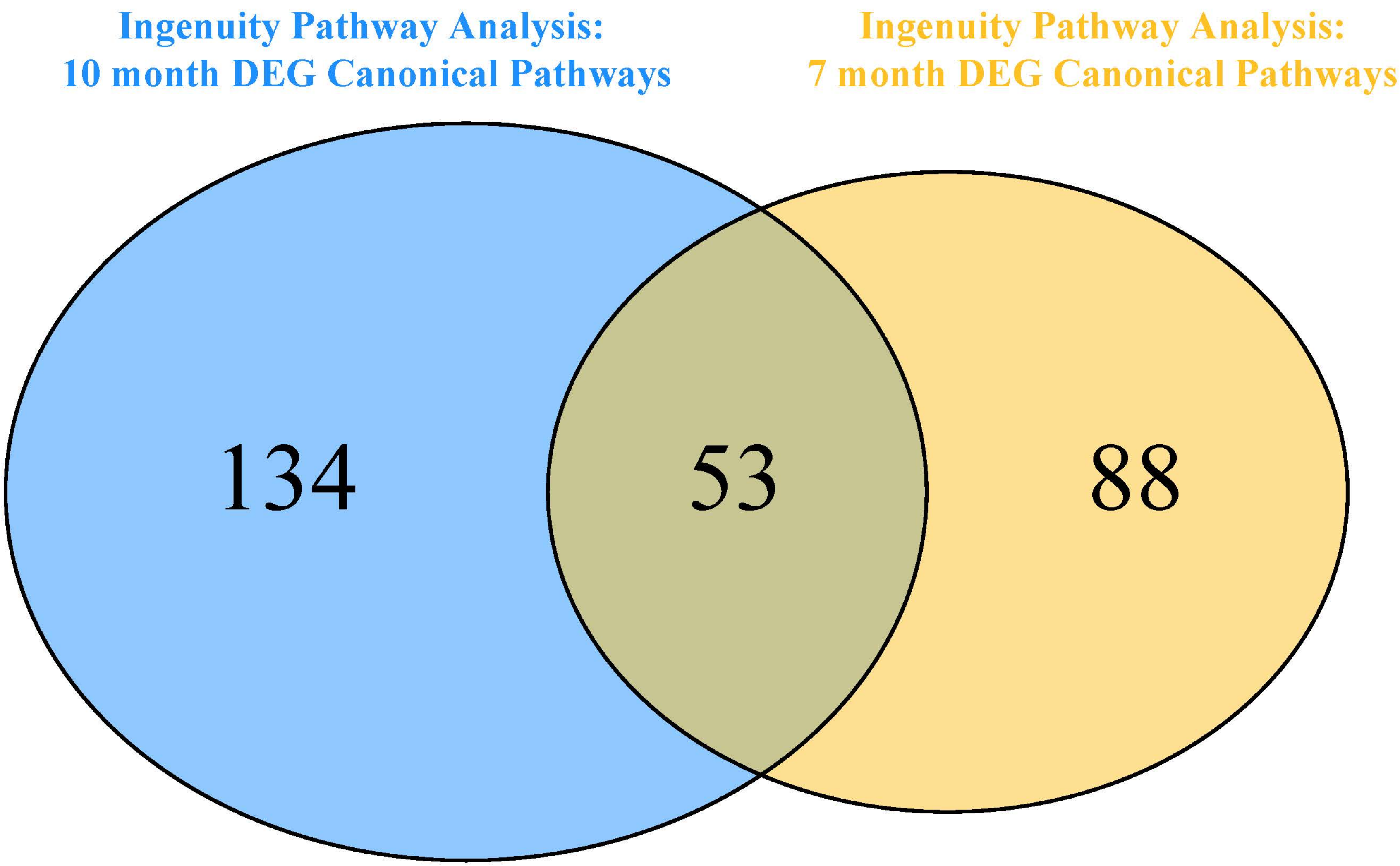
Ingenuity Pathway Analysis:
10 month DEG Canonical Pathways
Ingenuity Pathway Analysis:
7 month DEG Canonical Pathways
134
53
88

Supplementary Figure 5. Venn Diagram of Ingenuity Pathway Analysis Canonical Pathway Results From 7 and 10 Month Differentially Expressed Genes. Differentially Expressed Genes (DEGs) identified at 7 or 10 months were processed by IPA and resulting canonical pathway lists were assessed for overlapping pathway names. The majority of both 7- and 10-month pathway lists were unique but 53 were significantly enriched at both time points.

Supplemental Figure 6. Parkinson's Signaling Pathway : 10 month DEGs

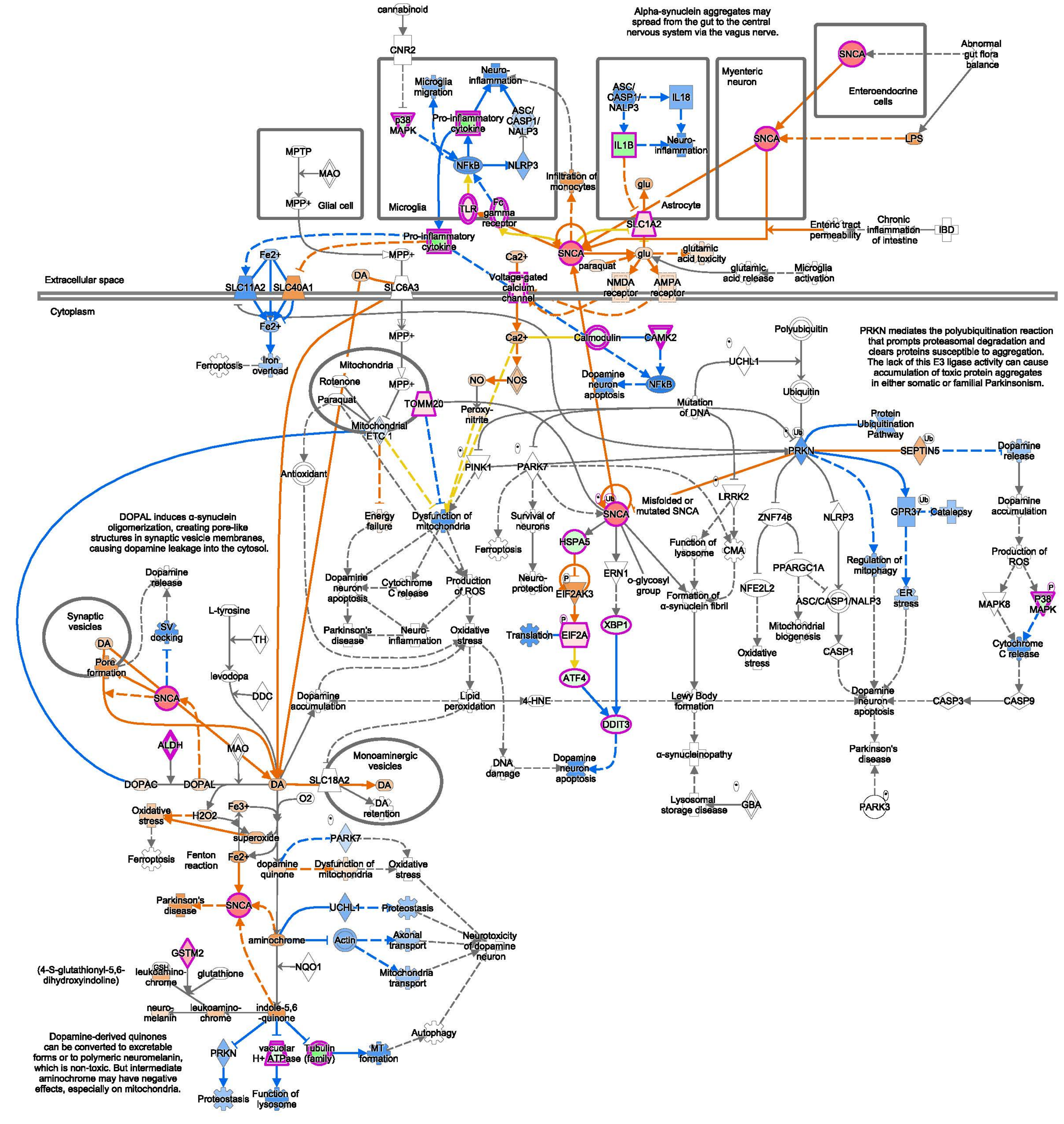

© 2000-2024 QIAGEN. All rights reserved.

Supplemental Figure 6. Ingenuity Pathway Analysis of 10-month Gene Expression: Enrichment of Parkinson's Signaling Pathway. Significant DEGs at 10 months were submitted to Qiagen's Ingenuity Pathway Analysis (IPA) for core analysis and enrichment canonical pathways. Identified DEGs involved in the pathway are outlined in purple and colored red if they are upregulated or green for down regulated genes. IPA also calculates a Z score for the pathway and individual interactions based on observed expression values to predict activation (orange) or inhibition (blue). Gray interactions were not able to be predicted and yellow interactions may be inconsistent between predicted and observed values. IPA likely predicts the pathway to be inhibited based on changes in multiple steps of the signaling pathways involved in apoptosis of dopaminergic neurons.

Supplemental Figure 7. Pyroptosis Signaling Pathway : 10 month DEGs

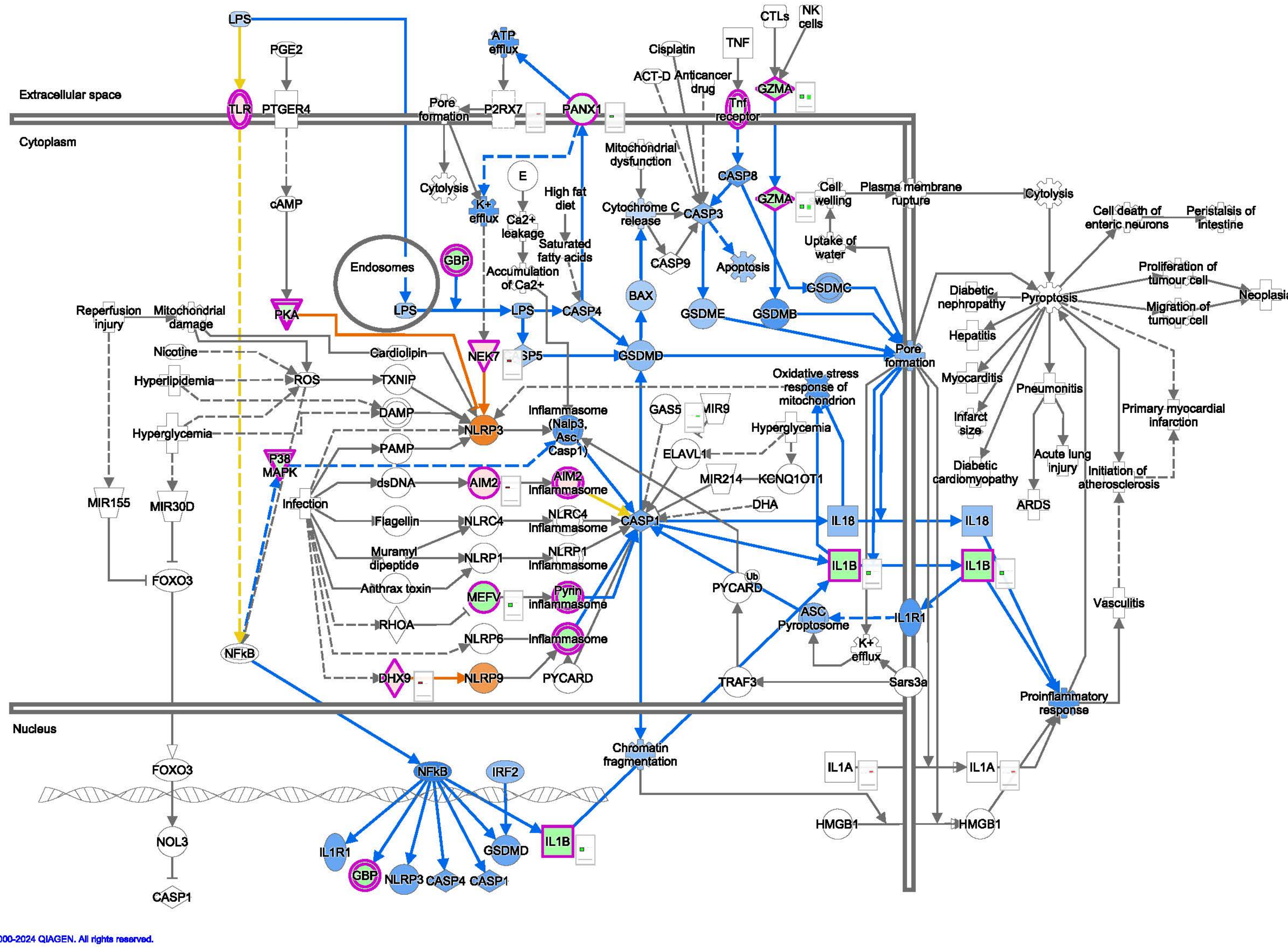

© 2000-2024 QIAGEN. All rights reserved.

Supplemental Figure 7. Ingenuity Pathway Analysis of 10-month Gene Expression: Enrichment of Pyroptosis Signaling Pathway. Significant DEGs at 7 months were submitted to Qiagen's Ingenuity Pathway Analysis (IPA) for core analysis and enrichment canonical pathways. Identified DEGs involved in the pathway are outlined in purple and colored red if they are upregulated or green for down regulated genes. IPA also calculates a Z score for the pathway and individual interactions based on observed expression values to predict activation (orange) or inhibition (blue). Gray interactions were not able to be predicted and yellow interactions may be inconsistent between predicted and observed values. IPA likely predicts pathway level inhibition of pyroptosis signaling based on decreased expression of inflammasome and proinflammatory genes.

Supplemental Figure 8. NOD1/2 Signaling Pathway : 7 month DEGs

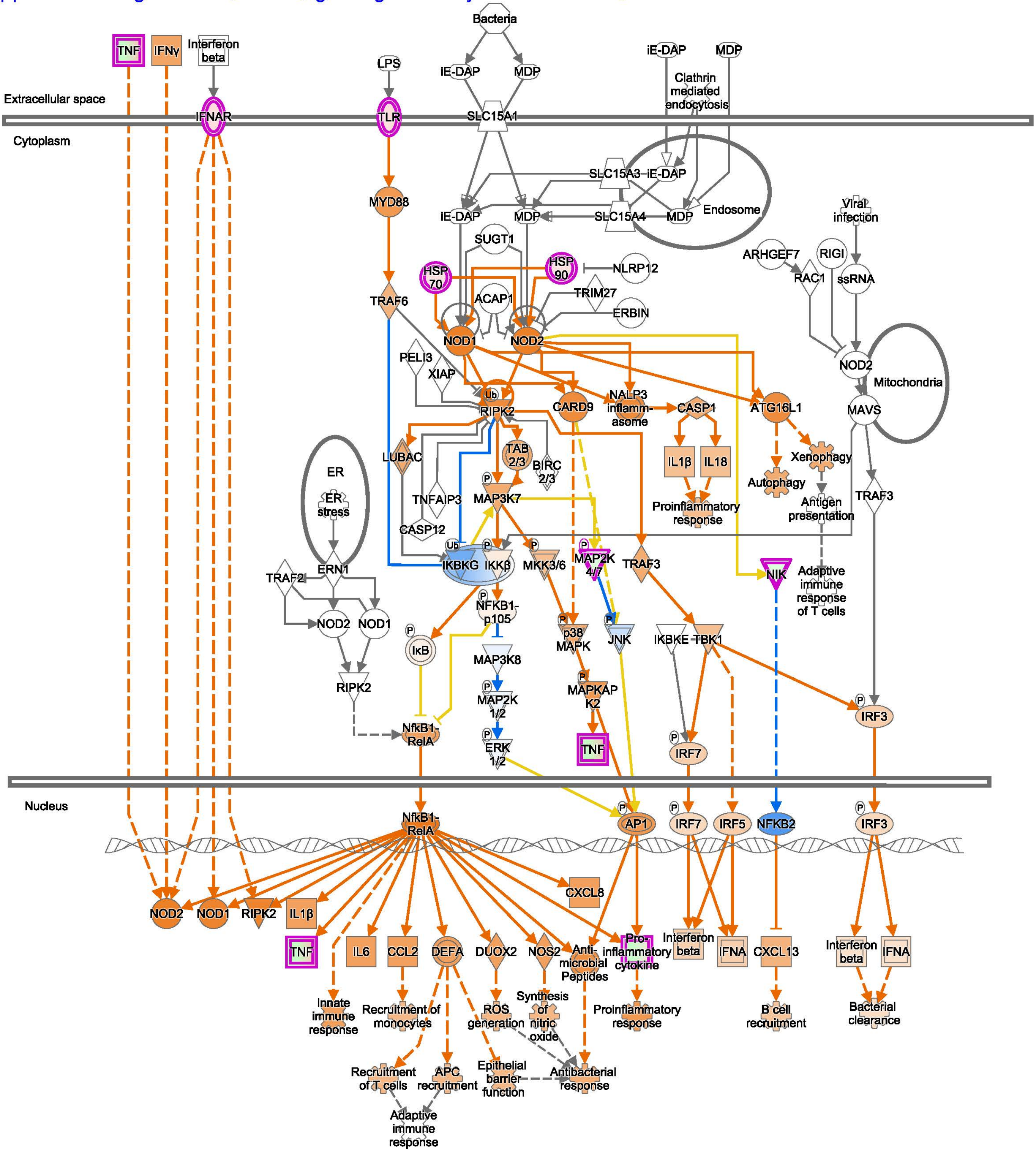

© 2000-2024 QIAGEN. All rights reserved.

Supplemental Figure 8. Ingenuity Pathway Analysis of 7-month Gene Expression: Enrichment of NOD1/2 Signaling Pathway. Significant DEGs at 7 months were submitted to Qiagen's Ingenuity Pathway Analysis (IPA) for core analysis and enrichment canonical pathways. Identified DEGs involved in the pathway are outlined in purple and colored red if they are upregulated or green for down regulated genes. IPA also calculates a Z score for the pathway and individual interactions based on observed expression values to predict activation (orange) or inhibition (blue). Gray interactions were not able to be predicted and yellow interactions may be inconsistent between predicted and observed values. IPA likely predicts activation at the pathway level based on increased expression of various TLRs, heat shock proteins, and other cytokine signaling genes.

Supplemental Figure 9. TREM1 Signaling : 7 month DEGs

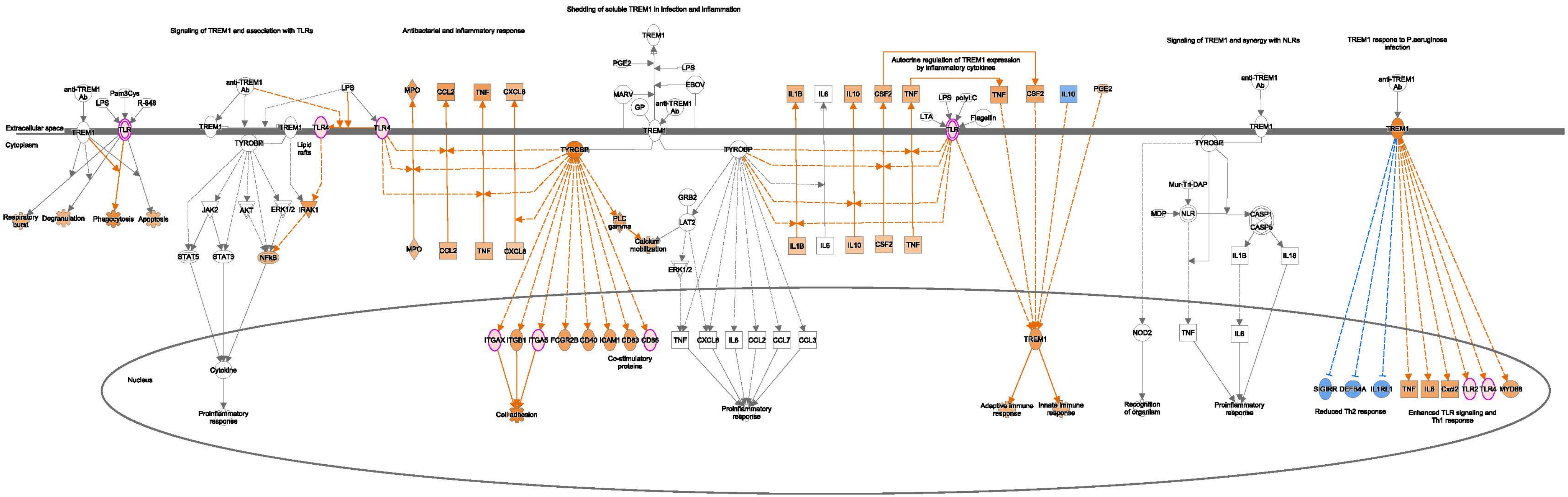

© 2000-2024 QIAGEN. All rights reserved.

Supplemental Figure 9. Ingenuity Pathway Analysis of 7-month Gene Expression: Enrichment of TREM1 Signaling. Significant DEGs at 7 months were submitted to Qiagen's Ingenuity Pathway Analysis (IPA) for core analysis and enrichment canonical pathways. TREM1 is involved in multiple aspects of the innate and adaptive immune response and acts to amplify signals from TLR and NLR proteins. Identified DEGs involved in the pathway are outlined in purple and colored red if they are upregulated or green for down regulated genes. IPA also calculates a Z score for the pathway and individual interactions based on observed expression values to predict activation (orange) or inhibition (blue). Gray interactions were not able to be predicted and yellow interactions may be inconsistent between predicted and observed values. IPA likely predicts activation at the pathway level based on increased expression of various TLRs and downstream genes such as ITGAX, ITGA5, and CD86.

Supplemental Figure 8. NOD1/2 Signaling Pathway : 10 month DEGs

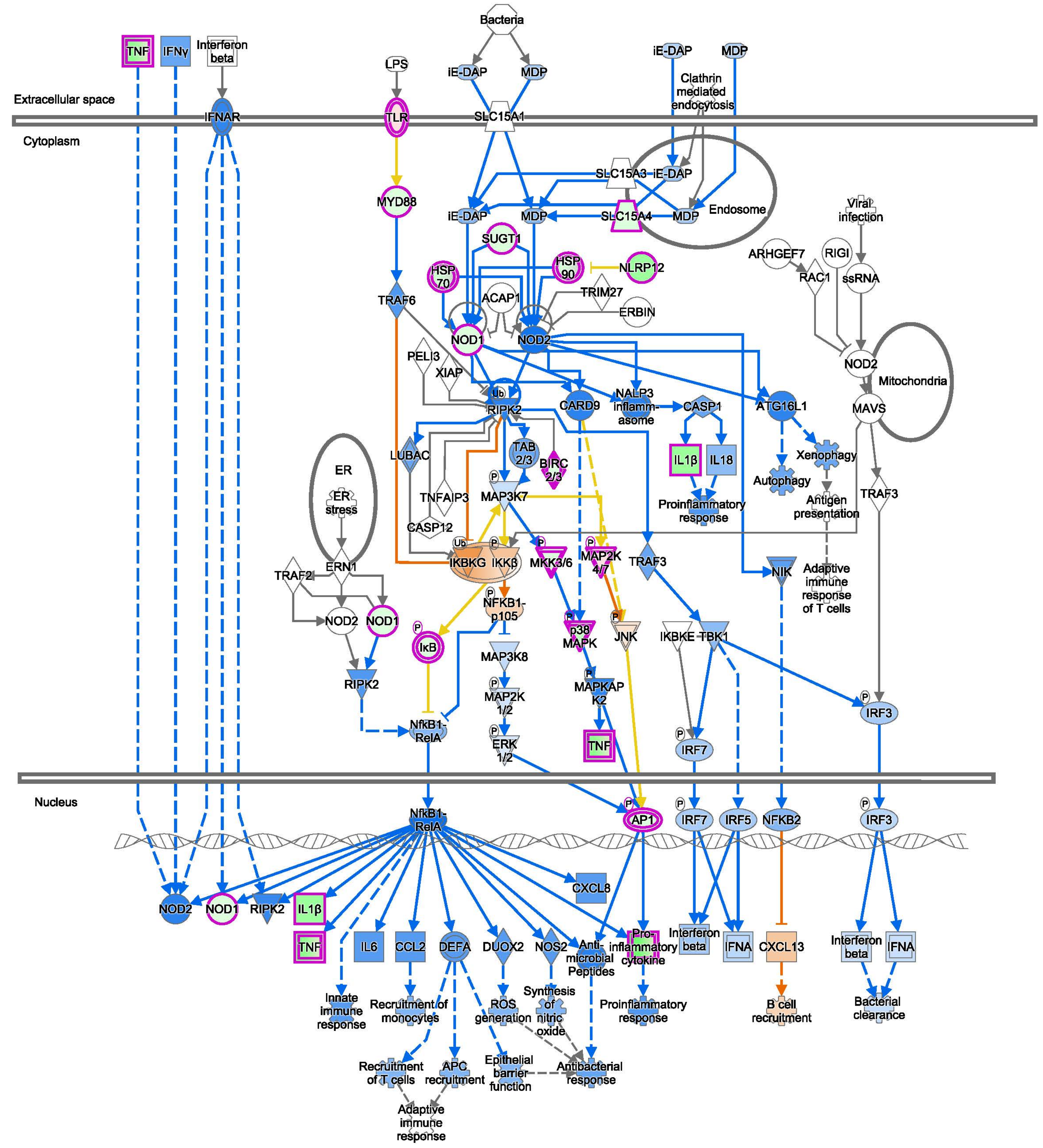

© 2000-2024 QIAGEN. All rights reserved.

Supplemental Figure 10. Ingenuity Pathway Analysis of 10-month Gene Expression: Enrichment of NOD 1/2 Signaling Pathway. Significant DEGs at 10 months were submitted to Qiagen's Ingenuity Pathway Analysis (IPA) for core analysis and enrichment canonical pathways. Identified DEGs involved in the pathway are outlined in purple and colored red if they are upregulated or green for down regulated genes. IPA also calculates a Z score for the pathway and individual interactions based on observed expression values to predict activation (orange) or inhibition (blue). Gray interactions were not able to be predicted and yellow interactions may be inconsistent between predicted and observed values. IPA likely predicts pathway level inhibition due to decreased expression of heat shock proteins, NOD1, and proinflammatory cytokines.

Supplemental Figure 11. TREM1 Signaling : 10 month DEGs

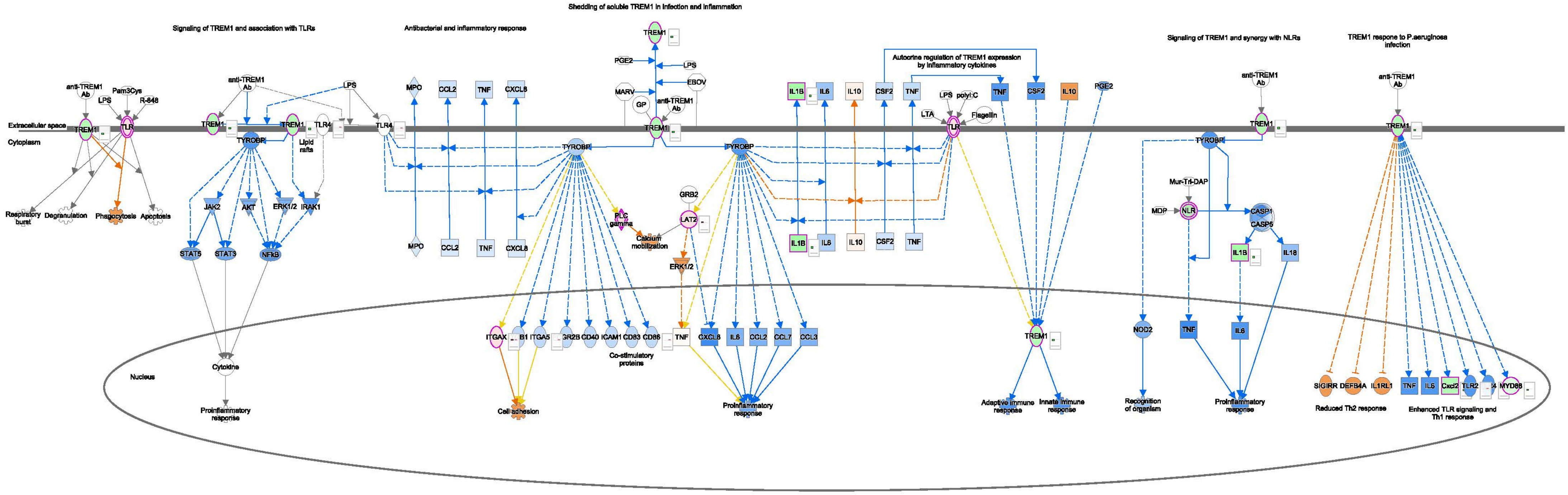

© 2000-2024 QIAGEN. All rights reserved.

Supplemental Figure 11. Ingenuity Pathway Analysis of 10-month Gene Expression: Enrichment of TREM1 Signaling. Significant DEGs at 10 months were submitted to Qiagen's Ingenuity Pathway Analysis (IPA) for core analysis and enrichment canonical pathways. TREM1 is involved in multiple aspects of the innate and adaptive immune response and acts to amplify signals from TLR and NLR proteins. Identified DEGs involved in the pathway are outlined in purple and colored red if they are upregulated or green for down regulated genes. IPA also calculates a Z score for the pathway and individual interactions based on observed expression values to predict activation (orange) or inhibition (blue). Gray interactions were not able to be predicted and yellow interactions may be inconsistent between predicted and observed values. IPA likely predicts pathway level inhibition due to decreased expression of TREM1, IL-1B, and the TLR-Th1 response.

Supplemental Figure 12. PPARα/RXRα Activation : 7 month DEGs

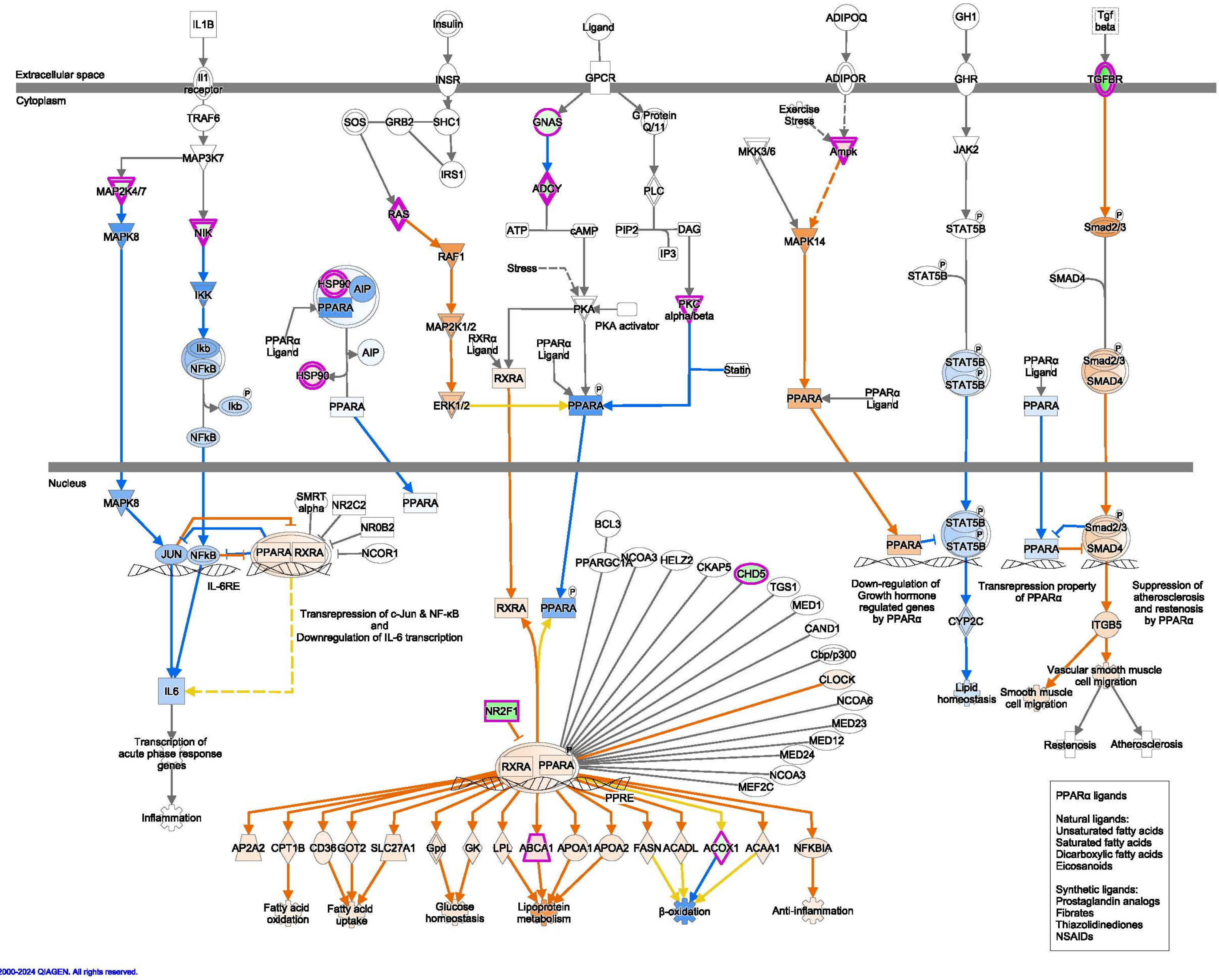

© 2000-2024 QIAGEN. All rights reserved.

Supplemental Figure 12. Ingenuity Pathway Analysis of 7-month Gene Expression: Enrichment of PPARα/RXRα Activation. Significant DEGs at 7 months were submitted to Qiagen's Ingenuity Pathway Analysis (IPA) for core analysis and enrichment canonical pathways. Identified DEGs involved in the pathway are outlined in purple and colored red if they are upregulated or green for down regulated genes. IPA also calculates a Z score for the pathway and individual interactions based on observed expression values to predict activation (orange) or inhibition (blue). Gray interactions were not able to be predicted and yellow interactions may be inconsistent between predicted and observed values.

Supplemental Figure 13. PPARα/RXRα Activation : 10 month DEGs

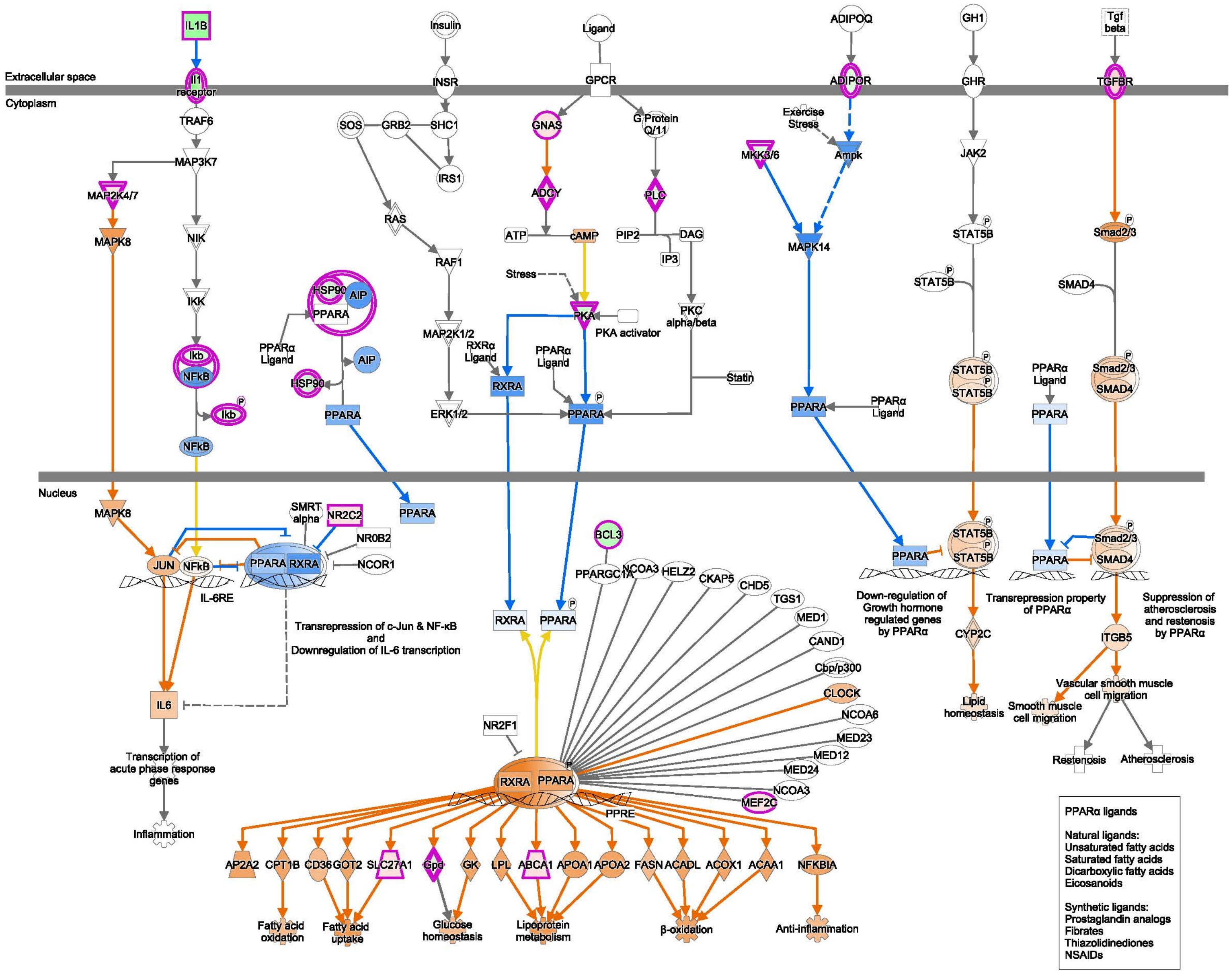

© 2000-2024 QIAGEN. All rights reserved.

Supplemental Figure 13. Ingenuity Pathway Analysis of 10-month Gene Expression: Enrichment of PPARα/RXRα Activation. Significant DEGs at 10 months were submitted to Qiagen's Ingenuity Pathway Analysis (IPA) for core analysis and enrichment canonical pathways. Identified DEGs involved in the pathway are outlined in purple and colored red if they are upregulated or green for down regulated genes. IPA also calculates a Z score for the pathway and individual interactions based on observed expression values to predict activation (orange) or inhibition (blue). Gray interactions were not able to be predicted and yellow interactions may be inconsistent between predicted and observed values.

Supplemental Figure 14. LXR/RXR Activation : 10 month DEGs

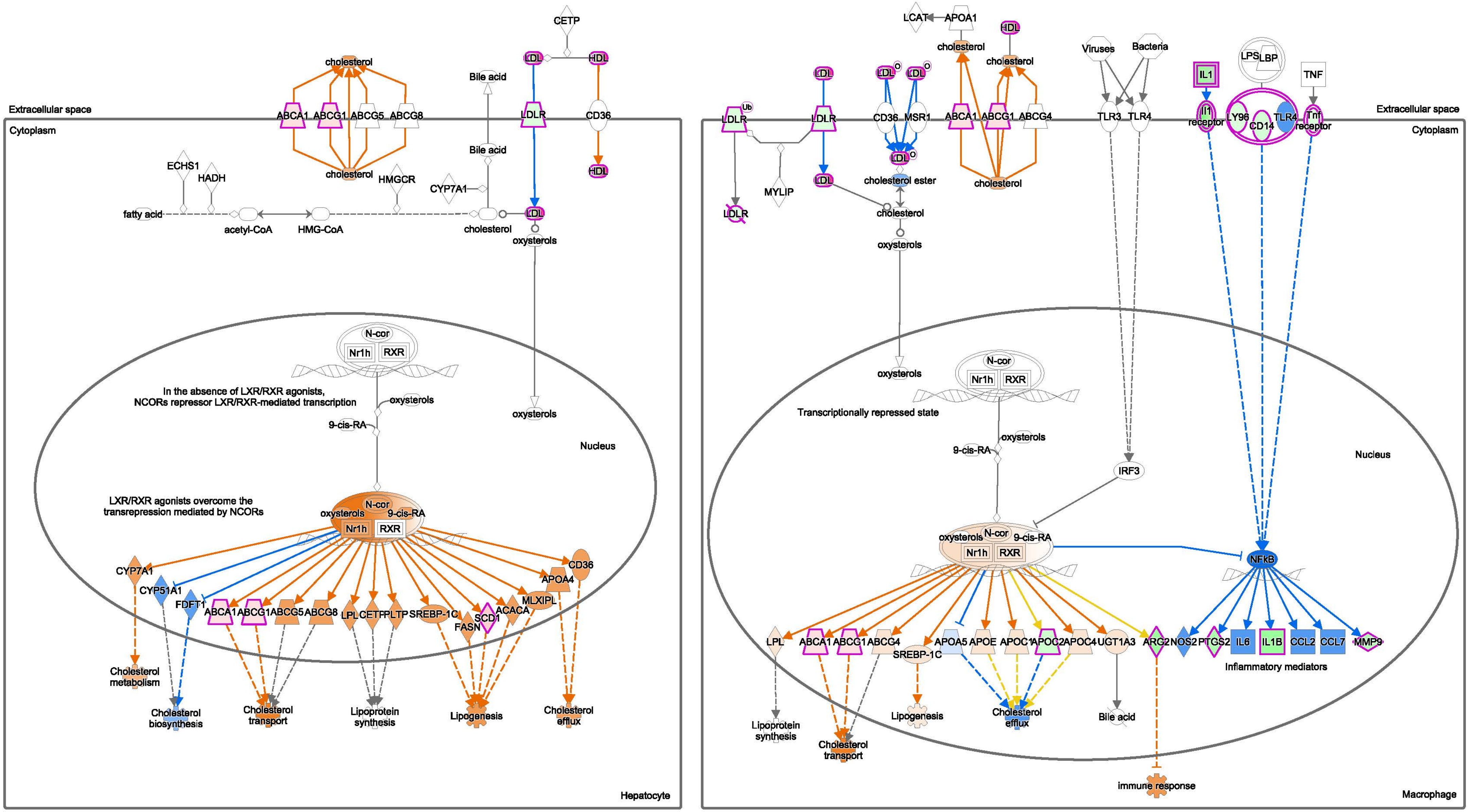

© 2000-2024 QIAGEN. All rights reserved.

Supplemental Figure 14. Ingenuity Pathway Analysis of 10-month Gene Expression: Enrichment of LXR/RXR Activation. Significant DEGs at 10 months were submitted to Qiagen's Ingenuity Pathway Analysis (IPA) for core analysis and enrichment canonical pathways. This diagram depicts the pathway in both hepatocytes and macrophages and for our data the microglia signaling is likely to be similar to the macrophage diagram. Identified DEGs involved in the pathway are outlined in purple and colored red if they are upregulated or green for down regulated genes. IPA also calculates a Z score for the pathway and individual interactions based on observed expression values to predict activation (orange) or inhibition (blue). Gray interactions were not able to be predicted and yellow interactions may be inconsistent between predicted and observed values. IPA predicts this pathway to be activated based on the increased expression of ABCA1 and ABCG1 as well as decreased expression of inflammatory mediators.

Supplemental Figure 15. Pathogen Induced Cytokine Storm Signaling : 10 month DEGs

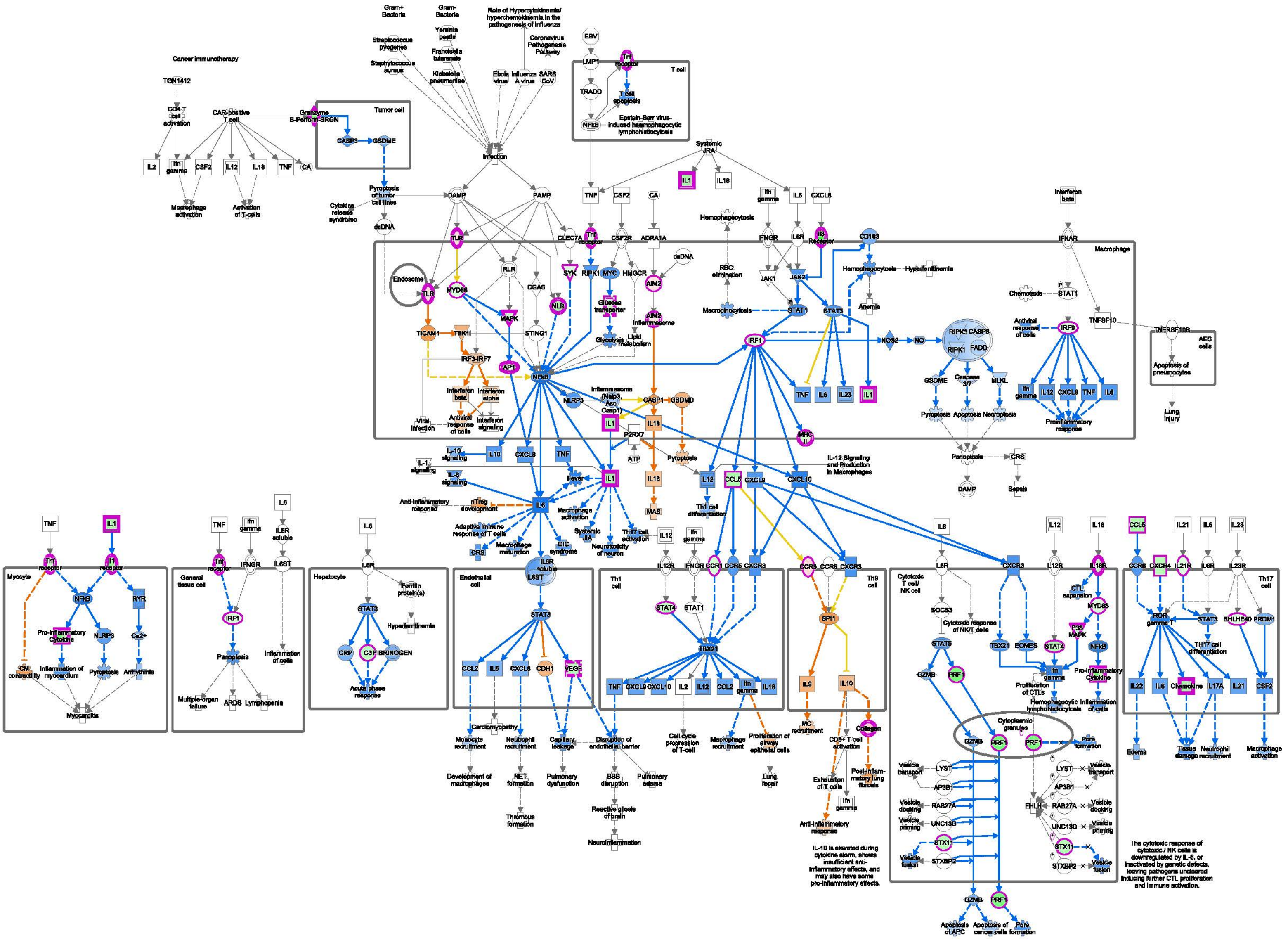

© 2000-2024 QIAGEN. All rights reserved.

Supplemental Figure 15. Ingenuity Pathway Analysis of 10-month Gene Expression: Enrichment of Pathogen Induced Cytokine Storm Signaling Pathway. Significant DEGs at 10 months were submitted to Qiagen's Ingenuity Pathway Analysis (IPA) for core analysis and enrichment canonical pathways. This pathway depicts changes in multiple cell types associated with immune signaling but our microglia derived data are likely to be most similar to the macrophage illustrated pathways. Identified DEGs involved in the pathway are outlined in purple and colored red if they are upregulated or green for down regulated genes. IPA also calculates a Z score for the pathway and individual interactions based on observed expression values to predict activation (orange) or inhibition (blue). Gray interactions were not able to be predicted and yellow interactions may be inconsistent between predicted and observed values. IPA predicts the cytokine storm pathway to be inhibited from the decreased expression of IL-1 and upstream NF-κB elements depicted despite a modest increase in TLRs and AIM2 inflammasome genes.
